# A systematic review of intracranial atherosclerosis epidemiology—the largest contributor to the global ischemic stroke burden?

**DOI:** 10.3389/fneur.2026.1866302

**Published:** 2026-09-18

**Authors:** Davor Pavlin-Premrl, Vincent Thijs, Peter J. Mitchell, Nawaf Yassi, Mark Parsons, Anna H. Balabanski, Julian Maingard, Hamed Asadi, Ashu Jhamb, Ali Khabaza, Adam Zheng, Aaron Gaekwad, Catherine Voutier, Bruce CV. Campbell

**Affiliations:** 1 Department of Medicine and Neurology, Melbourne Brain Center at the Royal Melbourne Hospital, University of Melbourne, Parkville, VIC, Australia; 2 Department of Neurology, Austin Hospital, Heidelberg, VIC, Australia; 3 Department of Radiology, St Vincent's Hospital, Fitzroy, VIC, Australia; 4 Florey Institute of Neuroscience and Mental Health, Melbourne, VIC, Australia; 5 Department of Radiology, Royal Melbourne Hospital, University of Melbourne, Parkville, VIC, Australia; 6 Population Health and Immunity Division, The Walter and Eliza Hall Institute of Medical Research, Parkville, VIC, Australia; 7 Health Sciences, Royal Melbourne Hospital, Melbourne, VIC, Australia; 8 Department of Neurology, Liverpool Hospital, University of New South Wales, Sydney, NSW, Australia; 9 Department of Neurology, Alfred Hospital, Melbourne, VIC, Australia; 10 Department of Radiology, Austin Hospital, Heidelberg, VIC, Australia

**Keywords:** diagnosis, epidemiology, ICAD = intracranial atherosclerotic disease, stroke, vessel wall imaging MRI

## Abstract

**Background:**

Intracranial atherosclerosis (ICAD) is commonly stated to be the largest contributor to the ischemic stroke burden worldwide. However, there has been little systematic examination of the epidemiology literature.

**Methods:**

PubMed, OVID-EMBASE and manual searches of references from retrieved literature were used to identify studies. Adult studies from 1990 onwards were included, with two reviewers independently reviewing titles and abstracts. Risk of bias was assessed using modified Newcastle-Ottawa Scale.

**Results:**

There were 138 studies included in the systematic review. Due to risk of bias and inconsistent definitions, there was low certainty in the findings of this systematic review. Moderate and high-quality studies based in Asia found that 8.8%−41.1% of stroke was due to ICAD, compared to 2.2%−11.1% in Europe and 7.5%−25% in North America. Moderate and high-quality studies investigating stroke recurrence in ICAD found a rate of 8%−28.3%. Moderate and high-quality studies investigating ICAD prevalence in unselected populations found a prevalence of 2.6%−58.5%. Moderate and high-quality studies found an ICAD prevalence of 5.4%−64.7% amongst stroke patients, compared with 15.5% found by a high-quality study from Europe.

**Conclusions:**

Epidemiological studies of ICAD demonstrate significant heterogeneity in design and risk of bias, leading to uncertainty regarding the true burden of this important disease. Nevertheless, current literature suggests that ICAD is at least as important as the other major etiologies in its contribution to the global ischemic stroke burden, highlighting the need for further research to inform therapeutic advances.

## Background

Intracranial atherosclerosis (ICAD) is commonly stated in the literature to be the commonest cause of stroke with the highest recurrence rate amongst patients from the most populous world regions, making it the largest contributor to the stroke burden worldwide (1). Despite a large body of work investigating the epidemiology of ICAD, there have been few reviews of this literature (2–4). Currently available reviews are not systematic (3), only investigate ICAD epidemiology in certain ethnicities (4) or only investigate limited epidemiological questions (2).

When considering ICAD epidemiology literature, it is important to delineate the major questions being examined. The most clinically relevant ICAD epidemiological questions are: the proportion of stroke due to ICAD and the recurrence rate amongst symptomatic ICAD patients. The answers to these questions help clinicians understand how frequently ICAD is responsible for stroke and guide the intensity of efforts to treat the disease. Other questions that are frequently investigated by ICAD epidemiology studies include the prevalence of asymptomatic ICAD in the general population, amongst stroke patients, and other high-risk groups.

There are several important sources of study design heterogeneity particular to ICAD epidemiology studies. It is important to consider in which world region the study was done, as it is generally considered that ICAD is more common amongst certain populations. How ICAD, symptomatic ICAD and recurrent symptomatic ICAD are defined is another important source of heterogeneity. Intracranial atherosclerosis is commonly defined using the method of measuring luminal stenosis laid out by the Warfarin-Aspirin Symptomatic Intracranial Disease (WASID) paper (5), variously at >30%, >50% and >70% stenosis. The WASID criteria do not include ICAD lesions where the vessel is positively remodeled (where the plaque does not impinge on the lumen). See Figure 1 for a visual demonstration of the issues in defining ICAD severity. How symptomatic ICAD is defined is another important source of heterogeneity. In many studies, a variant of the Trial of ORG 10172 in Acute Stroke Treatment (TOAST) (6) criteria is used and ICAD is considered to be the cause of cerebral ischaemia only if cardioembolism, extracranial carotid disease and small vessel ischaemia have been ruled out. This definition likely underestimates symptomatic ICAD as the disease shares the same risk factors as these causes and so frequently co-exists. Conversely, definitions of symptomatic ICAD that ignore other stroke etiologies will likely include bystander ICAD cases. How recurrent cerebrovascular events are defined is also important, with some papers only considering stroke as an outcome, and others bundling stroke and TIA together. Including TIA patients may mean the inclusion of some mimic patients.

**Figure 1:**
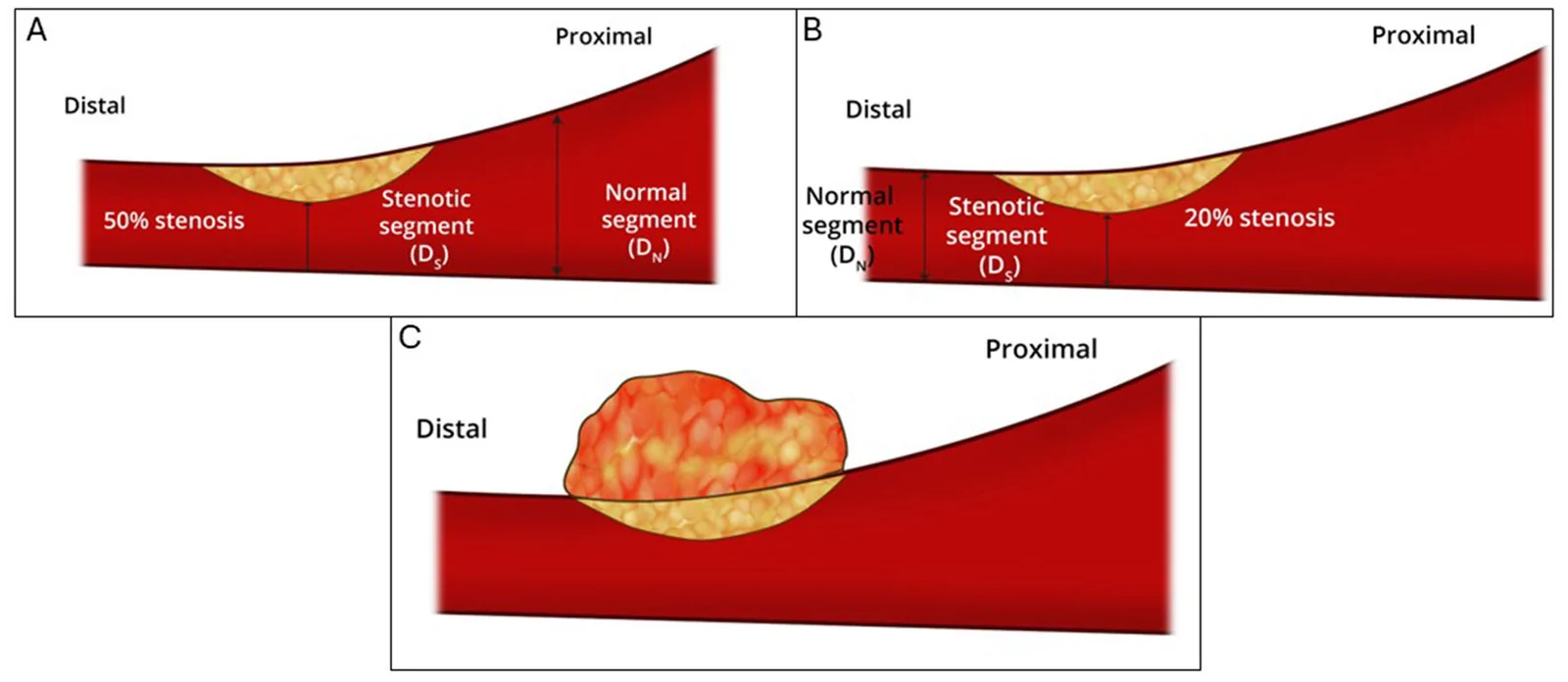
Measuring stenosis in ICAD. **(A)** Demonstrates measurement using the typical WASID criteria. **(B)** Demonstrates how the percentage stenosis can significantly change if the normal segment is taken to be the distal vessel rather than proximal vessel, as is convention when measuring carotid stenosis. **(C)** Demonstrates how the majority of plaque may not be identified if only the lumen is imaged. Together, these figures demonstrate the importance of imaging modality and clear definitions when investigating ICAD epidemiology.

Study quality is important to consider when evaluating ICAD epidemiology studies. Given the heterogeneity described above, clear definitions of ICAD and symptomatic ICAD are important. Another aspect of study quality is how ICAD was identified; the reference standard is autopsy with histological examination, but this is rarely available and not possible for non-fatal stroke. Direct subtraction angiogram (DSA) is the traditional reference standard as it provides excellent imaging of ICAD-related luminal stenosis (7). However, in recent times, MRI vessel-wall imaging has been shown to provide similar sensitivity/specificity for geometric evaluation of luminal stenosis compared to DSA. In addition, MRI black-blood imaging allows the identification of plaque that does not impinge on the lumen and positively remodels the vessel, a phenomenon that was previously only identifiable with pathological examination (8). The most common modalities for identifying ICAD in common clinical practice are CT angiogram, MRA TOF and transcranial Doppler. Of these, CT angiogram is the most sensitive, with 98% sensitivity compared with DSA, followed by MRA TOF, with 70% sensitivity compared with DSA (9), followed by transcranial Doppler, with 60% sensitivity compared with CTA (10). Other important factors when considering study quality are sample size and representativeness of the sample.

This study aimed to synthesize what is known and currently unknown about ICAD epidemiology to guide future research and health reform.

## Methods

Prior to commencing, the systematic review was registered on PROSPERO. Using methods in adherence with PRISMA guidelines, we searched PubMed, OVID-EMBASE and performed manual searches of references from retrieved literature to identify studies relating to ICAD epidemiology from 1990 to current (see appendix for detailed search strategy). Studies lacking sufficient information to derive prevalence and proportions were excluded. Using Covidence^®^, two reviewers independently validated the search strategies, screened titles and abstracts, and recorded reasons for rejection. Relevant articles underwent full-text screening.

Studies were grouped into studies evaluating the proportion of cerebrovascular disease due to ICAD, the rate of recurrent cerebrovascular events due to ICAD, the prevalence of ICAD in the general population and the prevalence of ICAD amongst stroke patients and other high-risk sub-groups. Quality was assessed using a modified version of the Newcastle-Ottawa scale that assessed representativeness of the sample, sample size, clear definition of patient groups and methods of ICAD identification (11). For studies of ICAD prevalence and proportion, studies that scored 5 points were deemed high-quality, studies that scored 3–4 were deemed moderate quality and studies that scored less than 3 were deemed poor quality. Studies that assessed recurrent cerebrovascular events were additionally assessed on outcome and follow-up. For these studies, those with 8–9 points were deemed as high quality, 4–7 points as moderate quality and less than 4 points as low quality. Other data extracted included the region/country study was performed, sample size, how ICAD was identified, how ICAD was defined, how symptomatic ICAD was defined, how cerebrovascular events were defined, proportion of patients with ICAD and proportion of patients with recurrent stroke. Studies were divided by epidemiological questions and extracted data tabulated. Matrices demonstrating heterogeneity in definitions and outcome measures were created. If meta-analysis was not possible due to heterogeneity, synthesis and reporting according to the Synthesis Without Meta-analysis (SWiM) reporting guidelines was planned. The certainty of the synthesized evidence for each primary outcome was formally assessed using the Grading of Recommendations Assessment, Development and Evaluation (GRADE) framework, starting at high certainty and then downgrading as appropriate. The overall body of evidence for each metric was evaluated across five specific domains: risk of bias, inconsistency, indirectness, imprecision, and publication bias.

Statistical analysis was performed with R statistical package (version 4.5.3).

## Results

There were 138 studies included in the systematic review, as summarized in the Figure 2. Of these, 32/138 (23%) studies investigate the proportion of stroke due to ICAD, 42/138 (30%) investigate stroke recurrence in ICAD, 18/138 (13%) investigate the prevalence of ICAD in healthy populations, 34/138 (25%) investigate the prevalence of ICAD amongst stroke patients and 20/138 (14%) investigate the prevalence of ICAD in other high-risk subgroups. There were multiple studies that answered multiple epidemiological questions. In terms of regions of origin, 77/138 (56%) were from Asia, 27/138 (20%) were from Europe, 21/138 (15%) were from North America, 10/138 (7%) were from other world regions and 3/138 (2%) were multi-regional. Heterogeneity in study methodology precluded meta-analysis.

**Figure 2:**
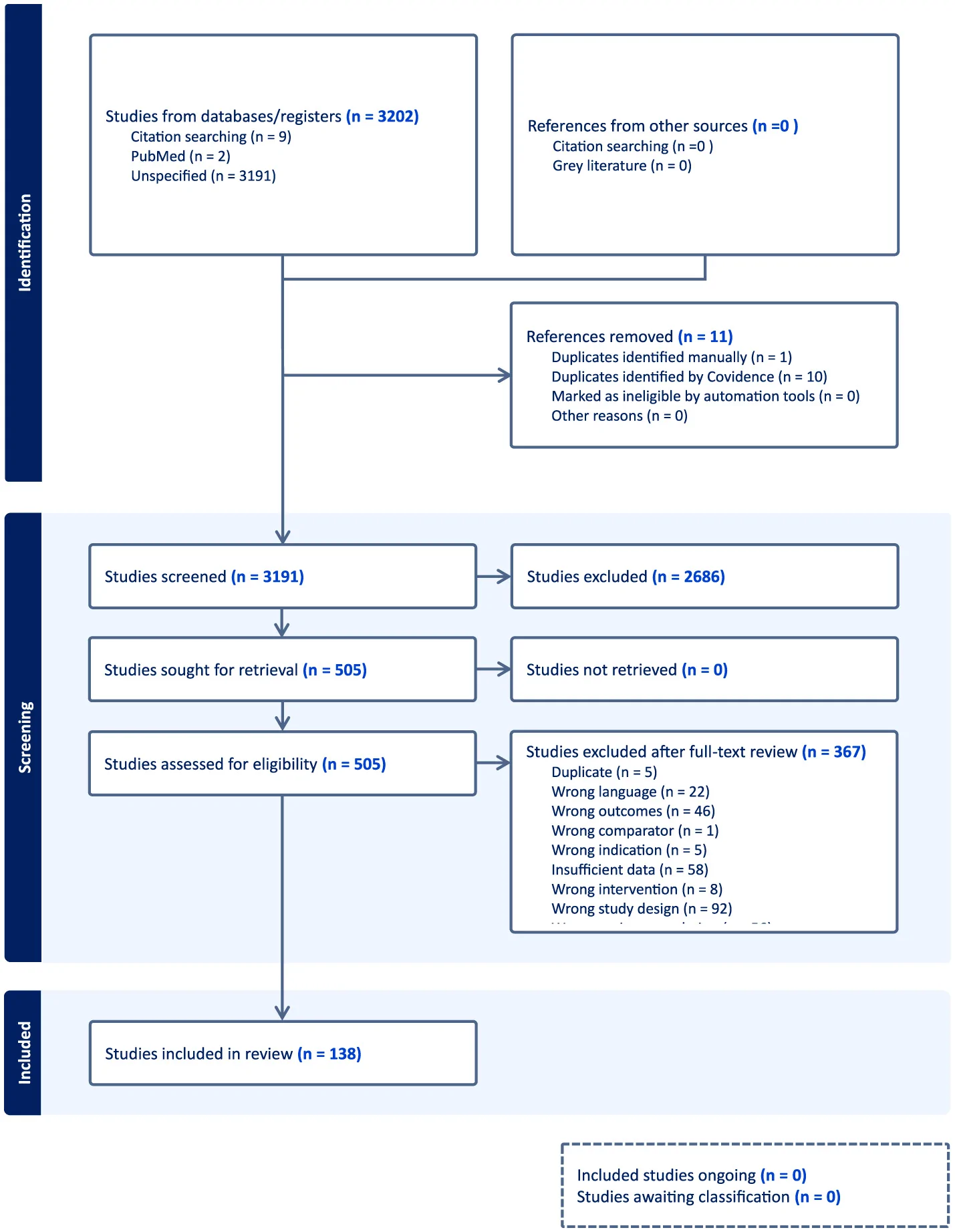
Study inclusion and exclusion flow diagram.

## Cerebrovascular disease due to ICAD

There were 32 studies investigating the proportion of cerebrovascular disease due to ICAD (Table 1). Given the understanding that ICAD is more common in certain regions, this portion of the narrative review has been divided into different world regions. High-quality and a selection of moderate quality studies have been summarized in Table 2, Figure 3 and Figure 4.

**Table 1:** Summary of findings.

Epidemiological question	Outcome	GRADE certainty
Proportion of stroke due to ICAD in Asia *Moderate and high quality studies*	8.8%−41.1%	Low
Proportion of stroke due to ICAD in Europe *Moderate and high quality studies*	2.2%−11.1%	Low
Proportion of stroke due to ICAD in North America *Moderate and high quality studies*	7.5%−25%	Low
Recurrence risk of same-territory stroke in ICAD *Moderate and high quality studies*	8%−28.3%	Low
Prevalence of ICAD amongst unselected populations *Moderate and high quality studies*	2.6%−58.5%	Low
Prevalence of ICAD amongst stroke patients *Moderate and high quality studies*	5.4%−64.7%	Low

**Figure 3:**
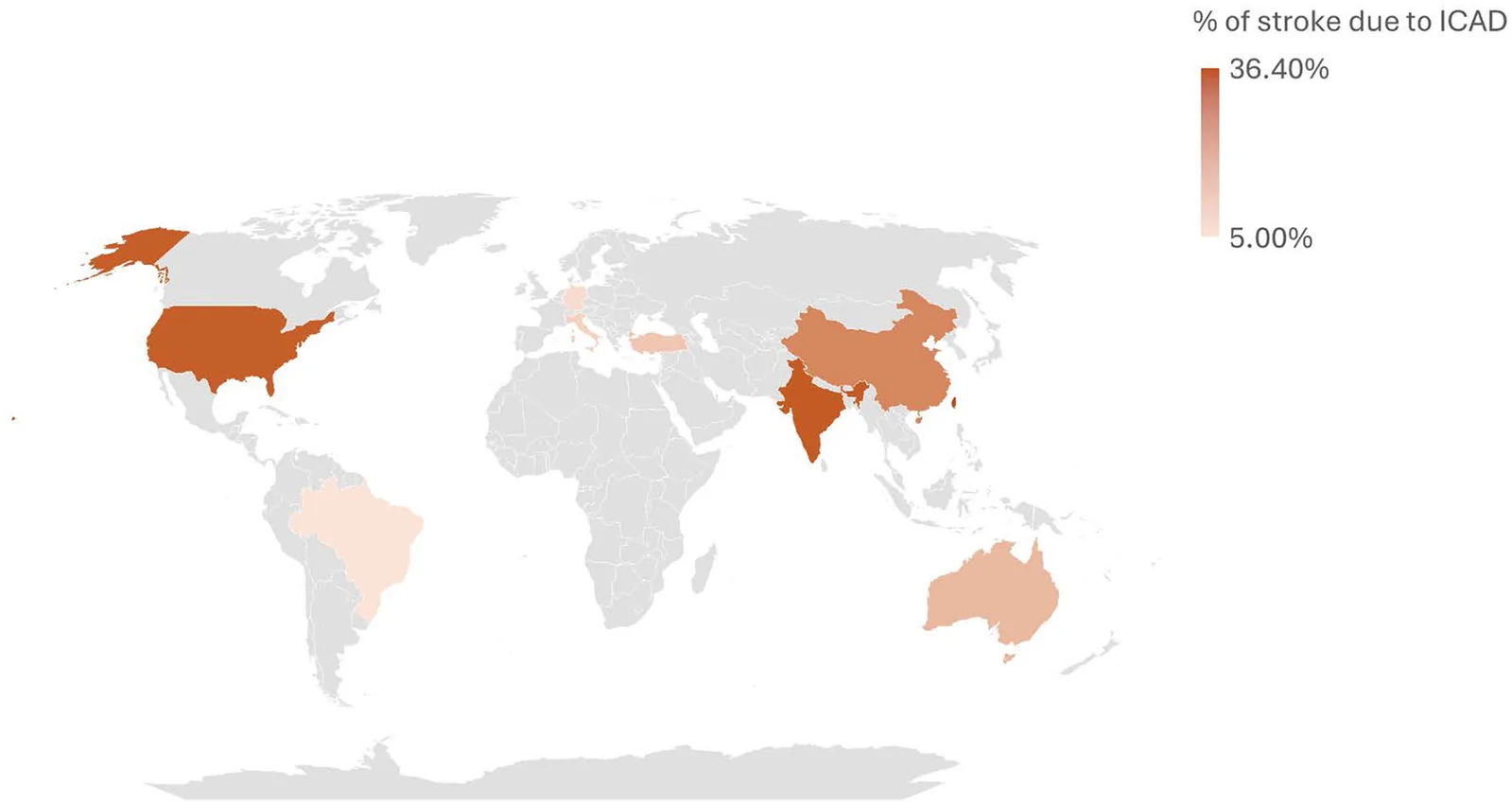
International distribution of percentage of stroke due to ICAD. If a country had multiple qualifying studies, the statistic from the highest quality study was selected. This map demonstrates that there are many countries where there is no available data. It also demonstrates that high rates of stroke due to ICAD is not necessarily limited to Asia.

**Figure 4:**
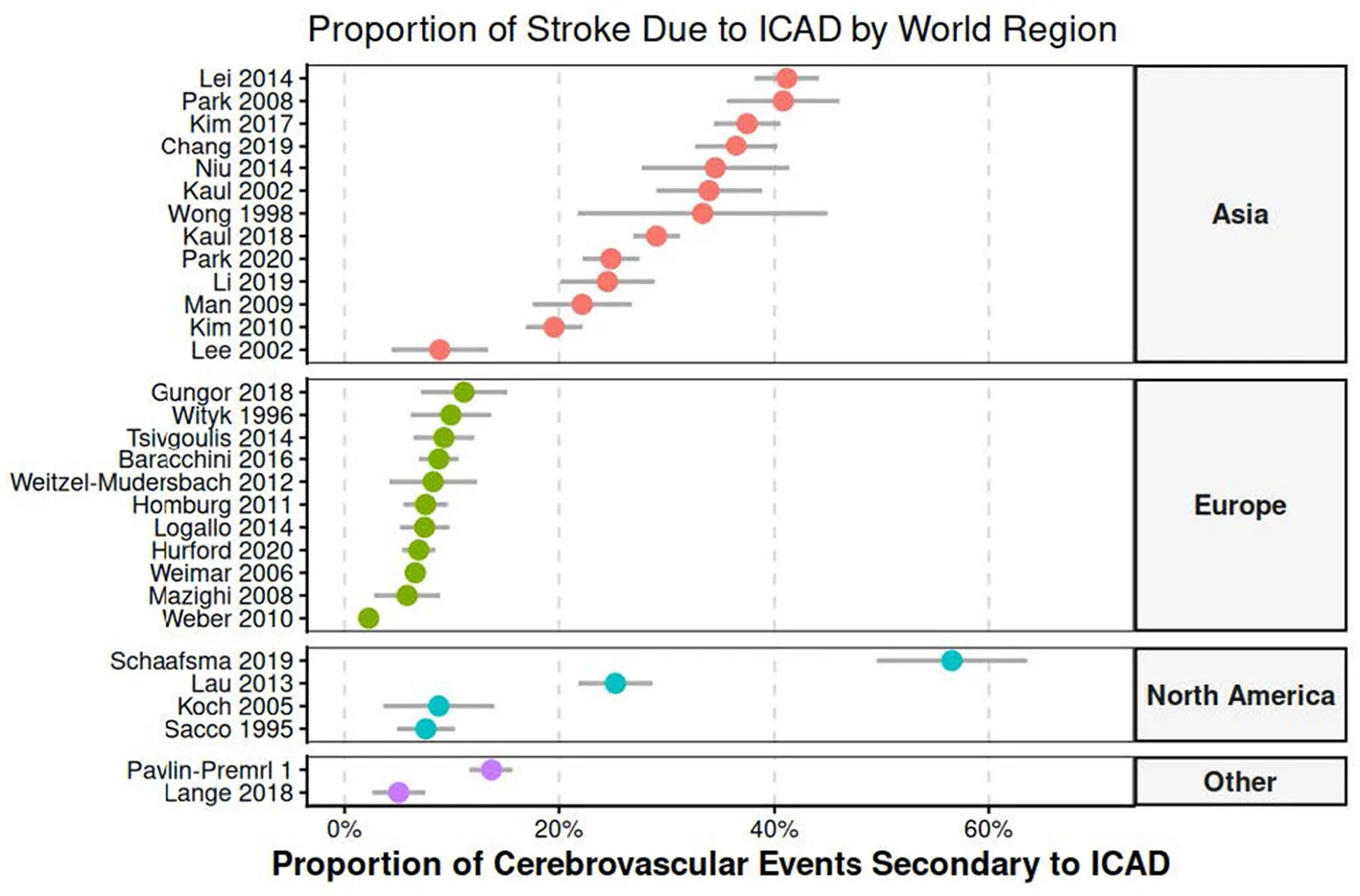
Studies investigating the proportion of stroke due to ICAD. Error bars are 95% confidence intervals.

**Table 2:** Proportion of cerebrovascular disease due to ICAD—selected papers.

References	Region	Country	Other notes on population	Newcastle-Ottawa scale score	Patient numbers	How ICAD was identified	ICAD definition	Symptomatic ICAD definition	Cerebrovascular event definition	Proportion of cerebrovascular events secondary to ICAD
Pavlin-Premrl, et al., (21)	Oceania	Australia		5	1,328	CTA	WASID, any stenosis or clinician judgement	Any same-vessel stroke or TIA	Stroke only	Stroke: (14%) 181/1,328
Chang, et al., (12)	Asia	Taiwan		4	686	MRI TOF	WASID, any stenosis	Any same-vessel stroke or TIA	Stroke and TIA	Stroke and TIA (not reported separately): 36.4% (250/686)
Park, et al., (14)	Asia	Korea	Korean patients admitted with acute ischaemic stroke, who underwent MRI/MRA	4	1,220	MRI TOF	WASID, >50%	Modified TOAST criteria	Stroke only	Stroke:24.8% (303/1,220)
Mazighi, et al., (15)	Europe	France	Autopsy study—fatal stroke only	4	259	Histopathology	Raymond Escourolle laboratory of neuropathology classification	Excludes cardioembolic stroke. Fatal stroke based on superimposed clot.	Stroke only	Stroke only: 5.8% (15/259)
Weber, et al., (16)	Europe	Germany		4	13,584	MRI TOF	WASID, >50%	modified TOAST criteria	Stroke and TIA	Stroke and TIA: 2.2% (304/13,584); not reported separately
Hurford, et al., (17)	Europe	UK		4	1,368	CTA or MRA TOF	WASID>50%	modified TOAST criteria	Stroke and TIA	Stroke and TIA: 6.9% (94/1,368); not reported separately
Lau, et al., (18)	North America	United States		5	697	CTA	WASID, any stenosis	Modified TOAST criteria	Stroke only	Stroke: 25% (176/697)
Schaafsma, et al., (19)	North America	Canada	Patients referred for vessel-wall MRI imaging imaging to clarify stroke etiology	3	205	CTA, DSA, MRI TOF, MRI VWI	MRI VWI reported by panel of neuroradiologists	modified TOAST	Stroke and TIA	Stroke and TIA: 57% (116/205) with MRI VWI
Sacco, et al., (20)	North America	United States		4	438	TCD	Standardized TCD criteria	Modified TOAST criteria	Stroke only	Total: 7.5% (33/438); – White: 1.2% (1/82); Black: 6.5% (10/155); Hispanic: 10.7% (21/197)
Lange, et al., (22)	South America	Brazil		4	359	MRI TOF, CTA or DSA	WASID, >50%	Modified TOAST criteria	Stroke only	Stroke: 5% (18/359)

## Asia

We found low-certainty evidence that 8.8% to 41.1% of stroke/TIA cases in Asia were due to ICAD. This was based on 11 moderate quality studies as there were no high-quality studies. As demonstrated in Table 2, these studies are quite heterogenous with variation in imaging methodology used and inclusion of TIAs creating risk of bias. There was also inconsistency in the definitions of ICAD and symptomatic ICAD. Chang et al., (12) performed a Taiwan-based study into stroke and TIA patients and found that 36.4% (250/686) of cases were due to ICAD. MRI TOF was used to identify cases, any percent stenosis with WASID was included and any same-vessel stroke was considered symptomatic ICAD (12). A Korean based study by Park et al., (13) found that 24.8% of stroke was due to ICAD. MRI TOF was used to identify patients. WASID criteria with >50% stenosis was used to define ICAD, and TOAST criteria were used to define symptomatic ICAD (14).

## Europe

We found low-certainty evidence that 2.2 to 11.1% of stroke/TIA cases in Europe were due to ICAD. There were 10 moderate quality studies as there were no high-quality studies. As demonstrated in Table 2, these studies were heterogenous with variation in imaging modality and the inclusion of TIAs creating risk of bias. There were also inconsistent definitions of ICAD and symptomatic ICAD. Particularly notable amongst these was Mazighi et al., (15), which used the reference standard of autopsy to show that 5.8% (15/259) of fatal stroke was due to ICAD. The study excluded cases where there were potential cardioembolic causes, and defined symptomatic ICAD as the presence of superimposed clot on ulcerated plaques (15). A large study by Weber et al., (16) performed in Germany found that only 2.2% (304/13,584) of patients' strokes/TIAs were due to ICAD. This study used MRI TOF, ICAD defined as stenosis >50% with WASID criteria and used modified TOAST criteria to define symptomatic ICAD (16). A UK study by Hurford et al., using predominantly MRI TOF found that 6.9% of stroke and TIA was due to ICAD. ICAD was defined using WASID criteria >50% and symptomatic ICAD was defined using modified TOAST criteria (17).

## North America

There was low certainty that ICAD contributed to 7.5 to 25% of stroke in North America. As seen in Table 2, there was one high quality study and five moderate quality studies that used inconsistent definitions and were at risk of bias due to the use of insensitive imaging methods and the bundling stroke and TIA patients together. Lau et al., (18) found that 25% of stroke was secondary to ICAD. Patients were investigated with CTA, ICAD was defined as any stenosis with WASID criteria and symptomatic ICAD was defined using the modified TOAST criteria (18). Schaafsma et al., performed a study investigating patients referred to their service for clarification of etiology in stroke and TIA patients. The study underscores the sensitivity of MRI vessel wall imaging (VWI) in identifying ICAD, with 57% (116/205) of stroke/TIA cases found to be due to ICAD when this modality was used, in comparison to 23% (48/205) when conventional imaging (MRI TOF, CTA, DSA) was used (19). A frequently-cited landmark study was the Northern Manhattan Stroke Study performed by Sacco et al., (20). The study used TCD to identify ICAD cases among stroke patients, defining symptomatic ICAD as cases where there was no alternative cardioembolic, extracranial carotid or small-vessel cause. The study found that 7.5% (33/438) of stroke was due to ICAD. It also found that there were significant differences between different ethnicities, with symptomatic ICAD present in 1.2% (1/82) of white patients, 6.5% (10/155) of black patients and 10.7% (21/197) of Hispanic patients (20).

## Other world regions

There was one high-quality study from Australia that found that 14% (181/1328) of ischaemic stroke was due to ICAD. Pavlin-Premrl et al., (2025) used CTA and modified WASID criteria to investigate the proportion of stroke cases due to ICAD (21). There was also one study of moderate quality study from Brazil. Lange et al., (22) performed a study in Brazil which found that 5% (18/359) of stroke was due to ICAD. MRI TOF, CTA or DSA were used to identify cases, defined as stenosis >50% as per WASID, with symptomatic ICAD defined using modified TOAST criteria (22).

## Recurrence risk of stroke in ICAD

There were 42 studies that investigated cerebrovascular event recurrence in symptomatic ICAD (see Table 3 for selected studies, Table 2 for the complete list and Figure 5 for a forest plot summary). It is important to distinguish that some of these studies investigated recurrence of same-territory stroke and some investigated recurrence of any stroke. There were 27 studies investigating recurrence of any stroke and 23 studies investigating recurrent same-territory ischemic stroke (with 8 studies investigating both questions). Amongst these studies, there were some important sources of quality differences and heterogeneity, including whether TIAs were included in the original cerebrovascular event and follow-up, the length of follow-up and the nature of medical therapy. Medical therapy was particularly impacted by the age of the study, as standard medical care for ICAD has progressed over the last decades.

**Figure 5:**
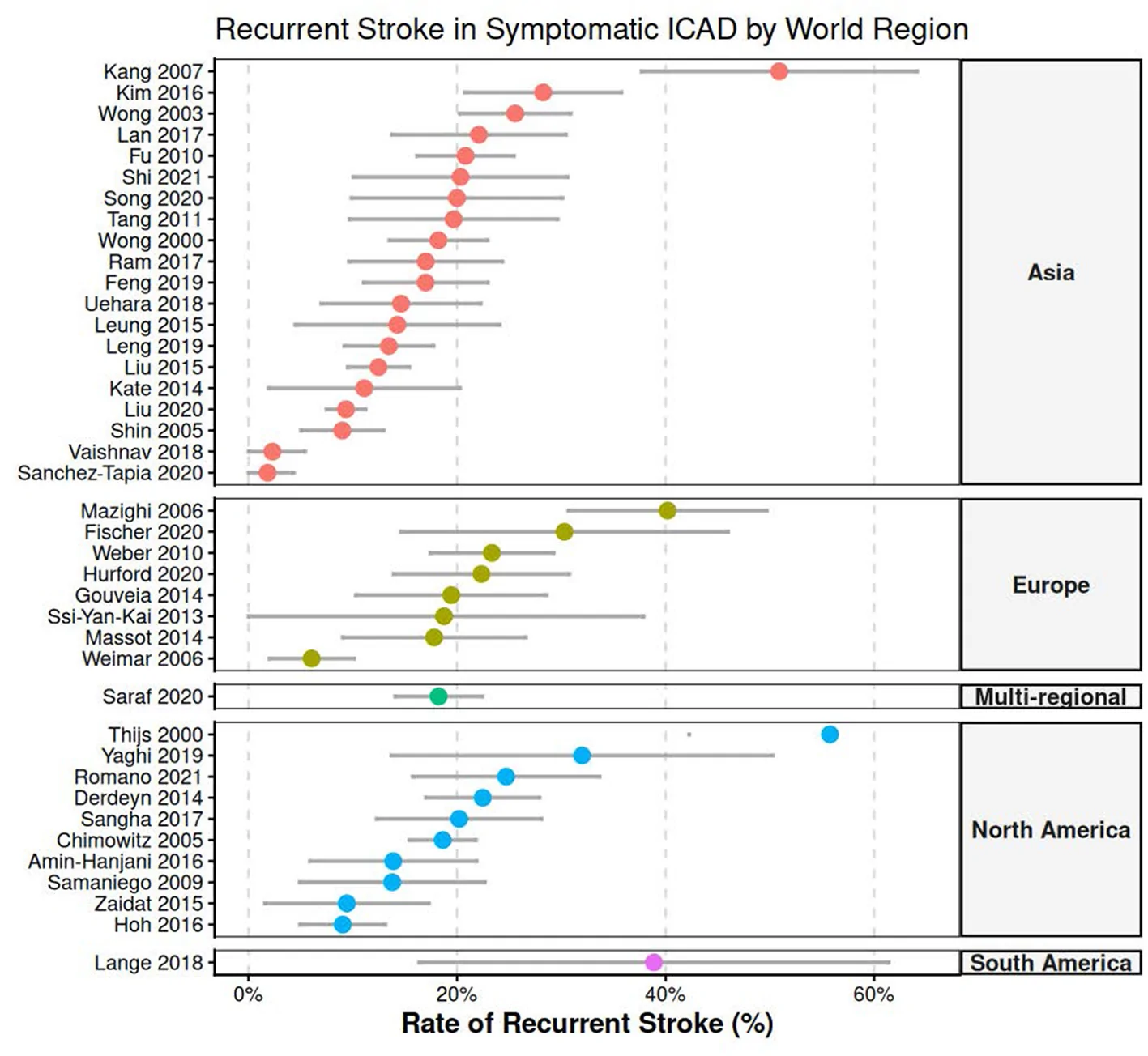
Studies investigating the rate of recurrent stroke in symptomatic ICAD. Error bars are 95% confidence intervals.

**Table 3:** Recurrent stroke in ICAD—selected papers.

References	Region	Country	Modified Newcastle-Ottawa scale score (out of 9)	Patient numbers	Patient population	How ICAD was identified	ICAD definition	Symptomatic ICAD definition	Medical therapy	Follow-up period	Recurrent cerebrovascular event rate
Derdeyn, et al., (23)	North America	United States	9	227	Patients enrolled in SAMMPRIS trial medical arm	DSA	WASID>70%	Excluded patients with cardioembolic or carotid sources	Aspirin for the duration of follow-up, clopidogrel for 90 days after enrolment. intensive management risk factor management.	2 year follow-up period: 11% (24/227) lost to follow-up	Recurrent same-territory stroke: 34/227 (15%); any recurrent stroke 23% (51/227)
Hurford, et al., (17)	Europe	UK	8	94	Stroke patients in Oxfordshire, UK	MRI TOF, CTA, DSA	WASID>50%	Any stroke or TIA in ICAD-affected vessel	Dual antiplatelets for 1 month, with single antiplatelet thereafter. Intensive risk factor management.	Median follow-up 2.8 years (IQR 1·5–4·6)	Recurrent same-territory stroke or TIA: cumulative risk 12% (11/94)
Romano, et al., (25)	North America	United States	8	105	Stroke and TIA patients	DSA, MRI TOF, CTA	WASID, >50%	Modified TOAST criteria	Intensive medical therapy based on the SAMMPRIS trial medical regimen	253 ± 31 days (mean ± SD), 2.9% (3/105) lost to follow-up	Recurrent same-territory stroke: 8.8% (2.7/100 patient-years); Same-territory infarct on MRI at 6–8 weeks: 24.7% (22/89)
Kang, et al., (26)	Asia	Korea	5	133	Stroke patients	MRI TOF	Measurement technique not specified, any stenosis	Any stroke in ICAD-affected vessel	Not specified	1 week	Any recurrent infarct on MRI at 1 week; 50.9% (28/55)
Kim, et al., (24)	Asia	Korea	8	138	Symptomatic intracranial atherosclerosis with stroke or TIA	MRI TOF followed by MRI VWI with vessel wall imaging	WASID, >50%	Modified TOAST criteria	At discretion of managing doctors (67.4% on dual antiplatelet therapy, 79% on statin)	Median 18 month follow-up (IQR 11–27 months)	Recurrent same-territory stroke 28.3% (39/138)

We found low-certainty evidence that same-territory recurrent stroke rates in ICAD are 8%−28.3%. Amongst high-quality and moderate quality studies there was risk of bias due to the use of insensitive imaging methods and sub-optimal medical therapy, and inconsistent definitions of ICAD and symptomatic ICAD. The highest quality study investigating recurrent stroke in ICAD was the medical arm of the SAMMPRIS study. Symptomatic ICAD was defined as stroke or TIA due to WASID criteria >70% stenosis, with more proximal causes excluded. Patients in the medical arm received dual antiplatelet therapy for 90 days, and aspirin thereafter, as well as intensive treatment and monitoring of vascular risk factors. Over a 2-year follow-up (with 11% lost to follow-up), the same-territory recurrence risk was 15% (34/227) and any recurrent stroke or death 23% (51/227) (23). Amongst moderate quality studies, the recurrent same territory stroke in ICAD was 8%−28.3%. Kim et al., (24) enrolled 138 Korean patients with symptomatic ICAD (stroke or TIA) using MRI VWI and WASID >50% criteria, and found that 28.3% had recurrent same-territory stroke. Median follow-up was 18 months and medical therapy at the discretion of the treating team, with most patients on dual antiplatelet therapy (24). Hurford et al., (17) followed up 94 patients with symptomatic ICAD (stroke or TIA) from Oxfordshire, UK and found that recurrent same-territory stroke or TIA cumulative risk was 12%, and recurrent stroke risk 5.5% over a median follow-up of 2.8 years. The cumulative risk of any stroke or death was 23.4% (21/94) over the same period (17).

Two studies have investigated recurrent covert stroke in symptomatic ICAD. Romano et al., (25) performed routine MRI DWI in patients with symptomatic ICAD (stroke or TIA) and found that 24.7% (22/89) had recurrent infarcts at 6–8 weeks. Kang et al., (26) performed MRI 1 week after presentation in patients with symptomatic ICAD and found that 50.9% (28/55) had a recurrent infarct on DWI.

## Prevalence of ICAD in unselected populations

There were 18 studies investigating ICAD in unselected populations (see Table 4 for highlighted studies and Table 3 for a complete list of studies). The majority of these studies were investigating ICAD prevalence in middle aged and older populations. Quality difference and heterogeneity was largely due to methods of ICAD identification.

**Table 4:** Prevalence of ICAD in unselected populations—selected studies.

References	Region	Country	Modified Newcastle-Ottawa scale score (total; of 5)	Population screened	Mean age	Patient numbers	How ICAD was identified	ICAD definition	Prevalence of ICAD
Qiao, et al., (27)	North America	United States	5	Black and white participants, recruited in middle age (45–64)	77.6 +/- 5.3 years	1,752	MRI VWI vessel wall imaging	WASID, >1%	Overall 36.4% (637/1,752); Black: 36.3% (188/518); White: 32.4% (400/1,234)
Suemoto, et al., (28)	South America	Brazil	5	Subjects dying from natural death of unknown cause who underwent autopsy	71.3 ± 11.7 years	661	Histopathology	>50% stenosis, other criteria not specified	58.5% (387/661)
Ahn et al., et al., (29)	Asia	Korea	4	Healthy subjects who underwent annual or biennial health checks	37.4	17,172	TCD	Standardized TCD criteria	3.55%
Jin, et al., (30)	Asia	China	4	Asymptomatic subjects aged >30 years	mean age 55.3	2,589	TCD	Standardized TCD criteria	4.7% (122/2,589)
Song, et al., (31)	Asia	China	4	General healthy population	Mean age 63 ± 13 years	3,640	TCD	Standardized TCD criteria	17.3% (629/3,640)
Park, et al., (32)	Asia	Korea	4	Asymptomatic volunteers for routine health examination	53	835	MRI TOF	WASID, >50%	3.0% (25/835)
Sun, et al., (33)	Asia	China	4	Asymptomatic subjects aged >40 years	57.6	2,019	TCD	Standardized TCD criteria	7.6% (153/2,019)

We found low-certainty evidence that the prevalence of ICAD in the general population is 2.6-58.5%. There were two important high-quality studies investigating ICAD prevalence in the general population. Qiao et al., (27) used MRI VWI imaging to investigate ICAD in an elderly USA population (mean age 77.6) and found that 36.4% of patients had ICAD. Suemoto et al., (28) found in a Brazilian autopsy study of patients who died of natural causes (mean age 71.3) that 58.5% had >50% obstruction of the intracranial arteries. Amongst moderate quality studies, Ahn et al., (29) investigated a relatively young Korean population (mean age 37.4) with TCD and found that 3.55% had ICAD. Jin et al., (30) investigated a slightly older (mean age 55.3) Chinese population with TCD and found 4.7% had ICAD. Song et al., performed a similar study in China with TCD, and found an ICAD prevalence of 17.3% in a population with an average age of 63 (31). In a Korean prevalence study, Park et al., used MRA and found that 3% of the general population had ICAD (mean age 53) (32). The Kongcun Town study performed in rural China used TCD to screen and found that 7.6% of the population had ICAD (mean age 57.6) (33).

## Prevalence of ICAD amongst high-risk subgroups

## Prevalence of ICAD amongst stroke patients

There were 34 studies that investigated the prevalence of ICAD amongst stroke patients (see Table 5 for select studies and Table 4 for the complete list). These studies have been distinguished from studies that investigate the proportion of cerebrovascular disease due to ICAD. The studies discussed here did not define symptomatic ICAD and did not limit reported ICAD in the affected vascular territory, therefore ICAD can be thought of as a marker of cerebrovascular health rather than the stroke etiology.

**Table 5:** Prevalence of ICAD amongst stroke patients.

References	Region	Country	Modified Newcastle-Ottawa scale score (total; of 5)	Patient numbers	Patient population details	How ICAD was identified	ICAD definition	Prevalence of ICAD
Zhang, et al., (34)	Asia	China	5	207	Stroke patients	CTA	WASID, >50%	64.7% (134/207)
Zhou, et al., (35)	Asia	China	5	1,101	Patients with first ischaemic stroke. Patients either Han Chinese or Zhuang Chinese.	CTA	WASID, any stenosis	Overall: 63.6% Han Chinese: 64.5% (483/749) Zhuang Chinese 61.6% (217/352).
Ovesen, et al., (36)	Europe	Denmark	5	652	Stroke and TIA patients	CTA	WASID, >30%	15.5% (101/652)
Leng, et al., (2)	Asia	China, UK	4	Total: 1,969, UK: 1,278, Hong Kong: 691	Stroke and TIA patients	MRI TOF, CTA, doppler	WASID, >50%	Total: 28% (554/1,969), UK 20% (257/1,278), Hong Kong 43% (297/691)
Markus, et al., (37)	Europe	UK	4	215	Stroke patients	MRI TOF, CTA, TCD	modified TOAST criteria	Total ICAD: 12% (26/215); amongst black patients: 17.9% (24/134); amongst white patients: 2.5% (2/81)
Moussouttas, et al., (38)	North America	United States	3	205	Stroke and TIA patients	TCD, CTA, MRA	Not reported	South Asian: 20% (20/99) European-American: 7% (7/106)

We found low-certainty evidence that the prevalence of ICAD amongst stroke patients is 5.4%−64.7%. Amongst high-quality studies from Asia, Zhang et al., (34) used CTA with WASID criteria >50%, and found that 64.7% of Chinese stroke patients had ICAD. Zhou et al., (35) also investigated Chinese stroke patients with CTA and found that 63.6% had ICAD (any stenosis, using WASID criteria). The prevalence was similar amongst Han Chinese (64.5%) and Zhuang Chinese (61.6%). Amongst high-quality evidence studies from Europe, Ovesen et al., (36) found that 15.5% of stroke patients from Denmark had ICAD on CTA. Leng et al., (2) performed a study investigating patients both in Asia and Europe. Using the same definitions and study protocols, they found that 20% of patients in Oxfordshire, UK had ICAD, compared with 43% of patients in Hong Kong, China. Markus et al., (37) also compared stroke patient ICAD prevalence between black (17.9%) and white (2.5%) patients in London. Another study that compared ICAD prevalence amongst stroke patients of different ethnicities was Moussouttas et al., (38). Performed in New Jersey, the study found that ICAD was more common amongst stroke patients of South Asian heritage (20%) than those of European-American heritage (7%). There are numerous studies investigating the prevalence of ICAD that demonstrate it is commonly found in patients in whom there is another potential cause. Lee et al., (39) found that 32.3% of patients with a potential cardioembolic cause of stroke had ICAD. Similarly, Park et al., (13) found that 24.8% of stroke patients with ICAD also had AF. Li et al., (40) found that 84.5% of patients with extracranial carotid atherosclerosis had ICAD. Palacio et al., (41) investigated patients with lacunar stroke and found that 17.3% had ICAD.

## Prevalence of ICAD amongst high-risk vascular groups

We identified 20 studies that investigate the prevalence of ICAD amongst high-risk subgroups. There are multiple studies that show that ICAD is common amongst patients with coronary artery disease and other high risk groups (Table 5). Alkan et al., found that 27.9% of patients being worked up for CABG had ICAD (42). According to Oh et al., TCD found that 4.4% of patients with coronary artery calcification had ICAD (43). Valaikiene et al., (44) found that 63.6% of patients with three-vessel or left stem coronary artery disease had ICAD. Jellinger et al., (45) found that 54% of patients with severe systemic atherosclerosis had ICAD.

There are multiple studies that demonstrate ICAD is common amongst patients with vascular risk factors. Hoshi et al., (46) found that 20.4% of patients referred for stroke primary prevention due to presence of vascular risk factors had ICAD. Park et al., (47) investigated a similar population and found that 8.8% had ICAD. Modesti et al., (48) investigated Chinese-heritage patients with type 2 diabetes and found that 17.7% had ICAD. Li et al., (49) found that 13.1% of patients with chronic kidney disease had ICAD.

## Discussion

### Is ICAD the commonest cause of stroke?

There is uncertainty about the true contribution of ICAD to the ischemic stroke burden as there is a paucity of high-quality studies answering this question. Furthermore, even amongst high and moderate-quality studies investigating symptomatic ICAD there was heterogeneity in imaging technique and definitions. Many studies may underestimate rates of symptomatic ICAD as they used insensitive imaging methods. Additionally, many excluded cases where there was an alternative potential cause. However, as this systematic review demonstrated there are significant rates of ICAD in patients with AF, extracranial carotid atherosclerosis and small vessel disease. It is likely that a proportion of cases of symptomatic ICAD have “bystander” atrial fibrillation or extracranial carotid atherosclerosis. Conversely, some studies may overestimate the proportion of stroke due to ICAD as they define any same-territory stroke as symptomatic ICAD. As demonstrated by the studies discussed in this review, presence of ICAD is very common amongst stroke and elderly patients. Much of it is likely not directly contributing to the ischemic stroke burden.

Despite the uncertainty created by these limitations, our review found that a significant proportion of stroke is likely due to ICAD (8.8% to 41.1% in Asia, 2.2 to 11.1% in Europe, 7.5 to 25% in North America). As a point of comparison, atrial fibrillation is thought to contribute to 10%−20% of the stroke burden, (50) carotid atherosclerosis approximately 15% (51) and small-vessel disease 25%−30% (52). ICAD is likely at least an equivalently important etiology to these more commonly considered etiologies, particularly given the majority of the world's population lives in Asia.

### Does ICAD have the highest risk of recurrent stroke?

There are relatively few high-quality studies that examine the recurrence of same-territory stroke in ICAD. Again, there was significant heterogeneity in definition of recurrent cerebrovascular events and ICAD. However, high and moderate quality studies show that same-territory cerebrovascular recurrence rate in ICAD was high despite medical therapy ranging from 8%−28.3%. In comparison, stroke recurrence in AF is ~3% with modern medical therapy (53) and >50% carotid atherosclerosis treated with endarterectomy has a 5 year risk of stroke of 4.1% (51). Furthermore, studies by Romano (25) and Kang (26) show that ICAD is associated with a high risk of recurrent covert infarcts, so the true recurrence rate of ischemic events is likely underestimated. Although additional research is needed to better understand the true frequency of recurrence, it is likely that symptomatic ICAD is associated with higher rates of recurrence than AF and carotid disease with current standard of care. This underscores the severity of ICAD as a pathology and the importance of more research into ICAD-specific secondary prevention.

### Does prevalence of ICAD vary by world region and ethnicity?

Intracranial atherosclerosis is commonly stated to be more common amongst Asian populations than European populations (54). Our systematic review supports this proposition, with high and moderate quality studies from Asia finding a higher proportion of stroke due to ICAD, more ICAD amongst stroke patients and more ICAD in the general population. It should again be noted, however, that there was significant heterogeneity between these studies. A notable exception is the study by Leng et al., which used the same study methods in a UK population and Hong Kong population, and found that ICAD was more common in the Hong Kong population (2). It should also be noted that the relationship between ethnicity and ICAD prevalence is likely more complex and granular than that which can be represented by comparing prevalence in different world regions.

Sacco et al., also compared white patients to black and Hispanic patients in the same Northern Manhattan cohort (20). Similarly, Markus et al., compared white and black patients in the same London cohort. Both studies found that white patients had the lowest rates of ICAD (37). These studies suggest that ICAD may have high prevalence in non-white populations outside of Asia. Unfortunately, there is very limited ICAD epidemiology data from Central America, South America and Africa to better understand prevalence in these regions. The reasons for variation in ICAD prevalence by region is currently poorly understood. Future high-quality epidemiological studies are needed to better understand the interplay between genetic and environmental factors.

### How common is ICAD in the general population?

Multiple high and moderate quality studies demonstrate that ICAD is common amongst middle-aged and elderly populations. This suggests that ICAD may be a useful marker of cerebrovascular health. It is also possible that these “asymptomatic” populations may actually have subclinical disease. Symptomatic ICAD has been associated with covert stroke, so it is possible that patients with asymptomatic ICAD also suffer covert cerebrovascular events. The connection between “asymptomatic” ICAD and cognitive outcomes is an important area for future population health research.

## Limitations and future recommendations

Our systematic review found that there is a large body of literature showing that ICAD is a common, severe disease. We also encountered frequent quality and heterogeneity issues which future epidemiology studies should ideally seek to avoid (see Table 6). This is consistent with research recently published by Navarro et al., who found that epidemiology studies of Asian ICAD populations suffer from significant heterogeneity, particularly regarding definitions and imaging modalities used (55). We support the authors' subsequent efforts to produce consensus definitions and imaging protocols (56, 57).

**Table 6:** Recommendations for future ICAD epidemiology papers.

Participants	>200
Imaging modality	•MRI vessel wall imaging •If not accessible, CT angiogram
Definition of ICAD	•WASID criteria •Multiple degrees of stenosis reported: any stenosis, >50%, >70% •Complete occlusion likely due to ICAD reported when possible •Positive remodeling due to ICAD reported when possible
Definition of symptomatic ICAD	•Cases should be divided into: • Likely symptomatic ICAD: >50% stenosis or high-risk plaque features with no competing causes • Possible symptomatic ICAD: >50% stenosis or high-risk plaque features with competing cause OR < 50% stenosis with no competing cause • Unlikely symptomatic ICAD: < 50% stenosis where stroke better explained by competing cause
Outcome reporting	•TIA and stroke should be reported separately

Many studies do not use sensitive methods of ICAD detection. Few studies use the most sensitive methods of autopsy and vessel-wall imaging. Future studies should use vessel-wall imaging if possible. CTA is the imaging modality of choice out of more generally accessible methods.

Another major issue identified by this systematic review is the heterogeneity in definitions used. This makes comparison between studies difficult, and prevents meta-analysis. There is heterogeneity in how ICAD is defined. Most studies use the WASID criteria, but define ICAD at different percentages of stenosis. Furthermore, there are ICAD lesions that are not identified by the WASID criteria, such as when there is positive remodeling or vessel occlusion. We do not believe that there is a particular definition of ICAD that is superior to others, but rather that each contains information that should ideally be reported. Thus we would favor an approach where data is reported using multiple definitions of ICAD.

The definition of symptomatic ICAD also varies. Many studies only define symptomatic ICAD where there are no other possible etiologies. This likely underestimates symptomatic ICAD as the disease is synergistic with other etiologies and shares risk factors. Conversely, defining all same-territory ICAD as symptomatic likely includes bystander lesions. We would favor an approach where the cases of same-territory ICAD are divided into likely cause, possible cause and unlikely cause, depending on the severity of the lesion and competing causes.

There is also heterogeneity in how cerebrovascular events are reported. Some studies report only stroke, others bundle stroke and TIA together. Ideally, data on stroke and TIA would be collected, but reported separately.

## Conclusion

Intracranial atherosclerosis epidemiology research has demonstrated that ICAD is at least as important as the other major stroke etiologies. Ongoing ICAD epidemiology research is needed to more accurately understand the burden of the disease, help prioritize ICAD research areas, assess population health and to better understand disease mechanisms. To achieve these aims, better standardization of study protocols and outcome reporting is needed.

## Data Availability

Publicly available datasets were analyzed in this study. This data can be found here: PubMed, OVID-EMBASE.
