# Supplementary material for: A systematic review of intracranial atherosclerosis epidemiology—the largest contributor to the global ischemic stroke burden?
Source: Front Neurol. 2026 Sep 18;17:1866302. doi: 10.3389/fneur.2026.1866302 (PMC13631950; doi:10.3389/fneur.2026.1866302)
Supplement: Supplementary file 1 [file Supplementary_file_1.docx]

# Supplementary materials:

## Search strategy:

| Population: Adults ≥ 18 | None specified |
| --- | --- |
| Outcome: Prevalence  #1 | **Controlled Subject Headings:**  "Epidemiology" [MeSH Subheading] OR  "epidemiologic studies" [MeSH Terms] OR  "Epidemiologic Methods"[MeSH Terms] OR  "Prevalence" [MeSH Terms] OR  "Population" [MeSH Terms] OR  "Population Groups" [MeSH Terms] OR  "Registries"[MeSH Terms] OR  "Observational study"[Publication Type]  **Synonyms (Title, Abstract, Keywords):**  Prevalence [TIAB] OR  Epidemiol*[TIAB] OR  Population-based[TIAB] OR  Population[TIAB] OR  Observational[TIAB] OR  Descriptive[TIAB] OR  Register*[TIAB] OR  Registry[TIAB] OR  Registries[TIAB] |
| Outcome: Recurrence  #2 | “Recurrence” [MeSH Term]  **Synonyms (Title, Abstract, Keywords):**  Recrudescence OR  Repeat OR  Deterioration |
| Outcome: Intracranial Atherosclerosis  #3 | **Controlled Subject Headings:**  “Intracranial Arteriosclerosis” [MeSH Heading]  **Synonyms (Title, Abstract, Keywords):**  Intracranial athero* [TIAB] OR  Intracranial arterio*[TIAB] OR  Cerebral arterio*[TIAB] OR  Cerebral athero*[TIAB] OR  Intracranial steno*[TIAB] OR |
| Search strings | #1 AND #3 OR #2 AND #3 |
| Limits | From 1990- |
| Notes on syntax | [TIAB] = includes title, abstract, keywords |

## Modified Newcastle-Ottawa Scale for prevalence studies:

Appendix. Modified Newcastle-Ottawa Scale (NOS) for quality assessment of the primary studies. - total of 5 points

(1) Representativeness of the sample:

1 point: truly representative of the average in the target population  * (all subjects or random sampling)

1 point: Somewhat representative of the average in the target population. * (nonrandom sampling)

0 points: selected group of users or no description

(2) Sample size:

1 point: Sample size was greater than 200 participants.

0 points: Sample size was less than 200 participants or a convenience sample.

(3) Identification of ICAD and non-ICAD:

1 point: Clear definitions of ICAD and symptomatic ICAD, non-ICAD patients drawn from same cohort as ICAD patients.

0 points: No description of non-ICAD patients, patients drawn from different cohort

(4) Identification/ascertainment of intracranial arterial stenosis (ICAS):

2 point: sensitive methods - autopsy, DSA, black blood, CTA

1 point: insensitive methods - MRA TOF, US

0 points: No description/non-standard imaging methods used

## Modified Newcastle-Ottawa Scale for recurrence studies:

Appendix. Modified Newcastle-Ottawa Scale (NOS) for quality assessment of the primary studies - total of 9 points.^1^

**Selection:**

(1) Representativeness of the sample:

1 point: truly representative of the average in the target population  * (all subjects or random sampling)

1 point: Somewhat representative of the average in the target population. * (nonrandom sampling)

0 points: selected group of users or no description

(2) Sample size:

1 point: Sample size was greater than 200 participants.

0 points: Sample size was less than 200 participants or a convenience sample.

(3) Identification of non-ICAD:

1 point: Comparability between ICAD and non-ICAD characteristics was established with clear definitions, non-ICAD patients drawn from same cohort as ICAD patients.

0 points: No description of non-ICAD patients, unclear definitions, patients drawn from different cohort

(4) Ascertainment of intracranial arterial stenosis (ICAS):

2 point: sensitive methods - autopsy, DSA, black blood, CTA

1 point: insensitive methods - MRA TOF, US

0 points: No description/non-standard imaging methods used

- If there are two methods used, whichever was the majority. If the proportion was not reported, then the least sensitive method

**Comparibility:**

(5) Comparability of cohorts on the basis of the design or analysis

1 point: same follow-up for cases and control

1 point: study controls for any additional factor

0 points: different follow-up for cases and controls

**Outcome: Ruled out interventional studies**

(6) Assessment of outcome

1 point: independent blind assessment

1 point: record linkage

0 points: self-report

0 points: no description

 (7) Was follow up long enough for outcomes to occur

1 point: at least 1 year

0 points: less than 1 year

(8) Adequacy of follow up of cohorts

1 point: complete follow-up, all subjects accounted for

1 point: subjects lost to follow up unlikely to introduce bias  (<20 % loss to follow up, or description of those lost) (1 point)

0 points: >20% loss to follow-up and no description of those lost

0 points: no statement

## Heterogeneity of studies:

### Proportion of stroke due to ICAD:

Amongst moderate and high-quality studies, the following matrices demonstrate the heterogeneity of studies.

Studies only including stroke patients:

|  | | Definition of symptomatic intracranial atherosclerosis | | | |
| --- | --- | --- | --- | --- | --- |
|  |  | modified TOAST criteria | Excludes cardioembolic stroke only | Any same-territory stroke | Not specified |
| Definition of intracranial atherosclerosis | WASID any stenosis, or cut-off<50% | 1 |  |  |  |
|  | WASID >50% stenosis | 3 | 1 | 1 | 1 |
|  | NASCET criteria | 1 |  |  |  |
|  | Histopathological definition |  | 1 |  |  |
|  | Standardised US criteria | 1 |  |  |  |
|  | No definition | 3 |  |  | 3 |

Studies including both stroke and transient ischaemic attack patients:

|  | | Definition of symptomatic intracranial atherosclerosis | | | |
| --- | --- | --- | --- | --- | --- |
|  |  | modified TOAST criteria | Excludes cardioembolic stroke only | Any same-territory stroke | Not specified |
| Definition of intracranial atherosclerosis | WASID any stenosis, or cut-off<50% | 2 |  | 2 |  |
|  | WASID >50% stenosis | 5 |  |  |  |
|  | NASCET criteria | 1 |  |  |  |
|  | Histopathological definition |  |  |  |  |
|  | Standardised US criteria |  |  |  |  |
|  | No definition | 2 |  |  |  |

### Recurrent stroke in ICAD:

Amongst moderate and high-quality studies, the following matrices demonstrate the heterogeneity of studies.

Studies only including stroke patients:

|  | | Definition of symptomatic intracranial atherosclerosis | | | |
| --- | --- | --- | --- | --- | --- |
|  |  | modified TOAST criteria | Excludes cardioembolic stroke only | Any same-territory stroke | Not specified |
| Definition of intracranial atherosclerosis | WASID any stenosis, or cut-off<50% | 3 |  | 1 | 1 |
|  | WASID >50% stenosis | 7 | 2 |  | 6 |
|  | NASCET criteria | 1 |  |  |  |
|  | Histopathological definition |  |  |  |  |
|  | Standardised US criteria |  |  |  |  |
|  | No definition | 1 |  | 1 |  |

Studies including both stroke and transient ischaemic attack patients:

|  | | Definition of symptomatic intracranial atherosclerosis | | | |
| --- | --- | --- | --- | --- | --- |
|  |  | modified TOAST criteria | Excludes cardioembolic stroke only | Any same-territory stroke | Not specified |
| Definition of intracranial atherosclerosis | WASID any stenosis, or cut-off<50% | 1 |  |  |  |
|  | WASID >50% stenosis | 2 | 2 | 3 | 3 |
|  | NASCET criteria |  |  | 1 |  |
|  | Histopathological definition |  |  |  |  |
|  | Standardised US criteria |  | 1 |  | 2 |
|  | No definition | 1 |  |  | 2 |

## GRADE Certainty Assessments:

|  | Outcome | Risk of Bias | Inconsistency | Indirectness | Imprecision | Publication Bias | Overall |
| --- | --- | --- | --- | --- | --- | --- | --- |
| Proportion of stroke due to ICAD in Asia  *Moderate and high quality studies* | 8.8-41.1% | Serious | Serious | Not serious | Not serious | Not serious | Low |
| Proportion of stroke due to ICAD in Europe  *Moderate and high quality studies* | 2.2-11.1% | Serious | Serious | Not serious | Not serious | Not serious | Low |
| Proportion of stroke due to ICAD in North America  *Moderate and high quality studies* | 7.5-25% | Serious | Serious | Not serious | Not serious | Not serious | Low |
| Recurrence risk of same-territory stroke in ICAD  *Moderate and high quality studies* | 8-28.3% | Serious | Serious | Not serious | Not serious | Not serious | Low |
| Prevalence of ICAD amongst unselected populations  *Moderate and high quality studies* | 2.6-58.5% | Serious | Serious | Not serious | Not serious | Not serious | Low |
| Prevalence of ICAD amongst stroke patients  *Moderate and high quality studies* | 8.8-64.7% | Serious | Serious | Not serious | Not serious | Not serious | Low |

## Data collection template:

Study ID

Title

Reviewer Name

Study ID

Title

Journal

Lead author

Year of Publication

Type of Publication

Country in which the study conducted

Have we captured all studies in the reference?

If no, the following studies have been added:

Study Objectives

Aim of study

Study design

World Region

Population description in detail

Age Group

Inclusion criteria

Exclusion criteria

Study Outcomes

How general population was identified if not a stroke population

How stroke patients were identified

How ICAD patients were identified

Study Period

Definition of Stroke

Definition of Transient Ischaemic Attack

Definition of Covert Stroke

Definition of Intracranial Atherosclerosis

Definition of Symptomatic Intracranial Atherosclerosis (incl. inclusion and exclusion)

Definition of recurrent cerebrovascular event - any

Definition of recurrent cerebrovascular event - same territory

Study funding sources

Possible conflicts of interest for study authors

Imaging Modalities/Methods Used in Study

Tesla and other details of protocol

MRI Details

Details of CT Angiogram Protocol

CT Angiogram Details

Details of TCD Protocol

Details of DSA Protocol

Details of Histopathology

Number of Readers

Blinding

Details of Medical Management

TIA included as recurrent cerebrovascular event

Covert infarct included as recurrent cerebrovascular event

Imaging required for recurrent cerebrovascular event

Follow up period

Method of identifying recurrent cerebrovascular event (ie clinical, routine, MRI)

Was recurrence in same vascular territory as index stroke

Patients numbers eligible/screened

Patient numbers recruited

Patient numbers recruited by sex Female (% and ratio)

Patient numbers recruited by ethnicity

Mean Age (IQR)

Patient numbers recruited by world region ( continent)

Patient numbers recruited by country

Patient numbers recruited by urban/rural background

Patient numbers recruited by neuroimaging

Patient numbers recruited by cerebrovascular event type - stroke or TIA

Total number of patients studied

Total number of patients with ICAD

Prevalence of ICAD amongst adults >18

Prevalence of ICAD amongst adults >18 by severity ( mild, mod, severe)

Prevalence of ICAD amongst stroke patients

Prevalence of ICAD amongst stroke patients by severity ( mild, mod, severe)

Proportion of stroke due to ICAD

Recurrence of cerebrovascular events in ICAD patients

Recurrence of cerebrovascular events in ICAD patients in same territory

Major limitations of the study

Assessment of bias according to Newcastle-Ottawa Scale (Prevalence study):

Score Representativeness of the sample: 1 point: truly representative of the average in the target population * (all subjects or random sampling) 1 point: Somewhat representative of the average in the target population. * (nonrandom sampling) 0 points: selected group of users or no description

Score Sample size: 1 point: Sample size was greater than 200 participants. 0 points: Sample size was less than 200 participants or a convenience sample.

Score Identification of non-ICAD: 1 point: Comparability between ICAD and non-ICAD characteristics was established, non-ICAD patients drawn from same cohort as ICAD patients. 0 points: No description of non-ICAD patients, patients drawn from different cohort

Score Identification/ascertainment of intracranial arterial stenosis (ICAS): 2 point: sensitive methods - autopsy, DSA, black blood, CTA 1 point: insensitive methods - MRA TOF, US 0 points: No description/non-standard imaging methods used

Score Total Score

Assessment of bias according to Newcastle-Ottawa Scale (Recurrence Study):

Score Representativeness of the sample: 1 point: truly representative of the average in the target population * (all subjects or random sampling) 1 point: Somewhat representative of the average in the target population. * (nonrandom sampling) 0 points: selected group of users or no description

Score Sample size: 1 point: Sample size was greater than 200 participants. 0 points: Sample size was less than 200 participants or a convenience sample.

Score Identification of non-ICAD: 1 point: Comparability between ICAD and non-ICAD characteristics was established, non-ICAD patients drawn from same cohort as ICAD patients. 0 points: No description of non-ICAD patients, patients drawn from different cohort

Score Ascertainment of intracranial arterial stenosis (ICAS): 2 point: sensitive methods - autopsy, DSA, black blood, CTA 1 point: insensitive methods - MRA TOF, US 0 points: No description/non-standard imaging methods used

Score Comparability of cohorts on the basis of the design or analysis a) same follow-up for cases and controls(1 point) b) study controls for any additional factor (1 point)

Score Assessment of outcome	a) independent blind assessment (1 point)	b) record linkage (1 point)	c) self report	d) no description

Score Was follow up long enough for outcomes to occur	a) yes (1+ year) (1 point)	b) no

Score Adequacy of follow up of cohorts	a) complete follow up - all subjects accounted for (1 point)	b) subjects lost to follow up unlikely to introduce bias - small number	lost - >80% follow up, or description of those lost) (1 point)	c) follow up rate <80% and no description of those lost d) no statement

Score Total Score

Will the paper make it into the narrative review

Contributions

| Table 1: Proportion of Stroke Due to ICAD | | | | | | | | | | |
| --- | --- | --- | --- | --- | --- | --- | --- | --- | --- | --- |
| Source | Region | Country | Other notes on population | Newcastle-Ottawa Scale Score | Patient numbers | How ICAD was identified | ICAD definition | Symptomatic ICAD definition | Cerebrovascular event definition | Proportion of cerebrovascular events secondary to ICAD |
| Pavlin-Premrl^2^ | Oceania | Australia |  | 5 | 1328 | CTA | WASID, any stenosis or clinician judgement | Any same-vessel stroke or TIA | Stroke only | Stroke: (14%) 181/1328 |
| Chang, 2019^3^ | Asia | Taiwan |  | 4 | 686 | MRI TOF | WASID, any stenosis | Any same-vessel stroke or TIA | Stroke and TIA | Stroke and TIA (not reported separately): 36.4% (250/686) |
| Kim, 2010^4^ | Asia | Korea |  | 4 | 1012 | MRI TOF | WASID, >50% | Modified TOAST criteria | Stroke (91.5%, 926/1012) and TIA (8.5%, 86/1012) | Stroke and TIA: 20% (198/1012); not reported separately |
| Li, 2019^5^ | Asia | China |  | 3 | 408 | CTA | Not reported | Not reported in detail. Other potential causes of stroke excluded. | Stroke only | Stroke: 24.5% (100/408) |
| Park, 2020^6^ | Asia | Korea | Korean patients admitted with acute ischaemic stroke, who underwent MRI/MRA | 4 | 1220 | MRI TOF | WASID, >50% | Modified TOAST criteria | Stroke only | Stroke:24.8% (303/1220) |
| Kaul, 2002^7^ | Asia | India |  | 3 | 392 | MRI TOF and TCD | Measurement technique not specified | Not specified | Stroke only | Stroke: 33.9% (133/392) |
| Kim, 2017^8^ | Asia | Korea |  | 3 | 1081 | MRI TOF | WASID, >50% | Modified TOAST criteria | Stroke and TIA, proportion of each not reported | Stroke and TIA: 21.2% (405/1081); not reported separately |
| Lee, 2002^9^ | Asia | Taiwan | Young stroke patients, aged 18-45 | 3 | 170 | DSA, MRI TOF | NASCET, >50% | Modified TOAST criteria | Stroke (91.3%, 155/170) and TIA (8.7%, 15/170) | Stroke and TIA: 8.8% (15/170); not reported separately |
| Lei, 2014^10^ | Asia | China | Chengdu Stroke Registry Project | 3 | 1196 | MRI TOF , CTA or DSA | Not reported | Not reported in detail, but excluded cardioembolic and "other obvious causes" | Stroke only | Stroke: 41.1% (492/1196) |
| Man, 2009^11^ | Asia | China |  | 3 | 343 | MRI TOF | WASID, any stenosis | Modified TOAST criteria | Stroke or TIA; proportion of each not reported | Stroke and TIA: 22.2% (76/343); not reported separately |
| Park, 2008^12^ | Asia | Korea |  | 3 | 370 | MRI TOF | Not reported | Modified TOAST criteria | Stroke only | Stroke: 40.8% (151/370) |
| Kaul, 2018^13^ | Asia | India |  | 2 | 2072 | MRI TOF | Measurement technique not reported | Modified TOAST criteria | Stroke only | Stroke: 29% (602/2072) |
| Niu, 2014^14^ | Asia | China | Young stroke patients, aged 15-49 | 2 | 197 | MRI TOF, CTA and TCD | Not reported | Modified TOAST criteria | Stroke only | Stroke: 34.5% (68/197) |
| Wong, 1998^15^ | Asia | China |  | 2 | 66 | TCD | Standardised TCD criteria | Stroke only; excludes cardioembolic stroke | Stroke only | Stroke: 33.3% (22/66) |
| Homburg, 2011^16^ | Europe | Netherlands |  | 4 | 786 | CTA | WASID, >30% | All same-vessel stroke. Did not exclude patients with potential cardioembolic or extracranial carotid causes. | Stroke and TIA | Stroke and TIA: 7.5% (59/786); not reported separately |
| Mazighi, 2008^17^ | Europe | France | Autopsy study - fatal stroke only | 4 | 259 | Histopathology | Raymond Escourolle Laboratory of Neuropathology classification | Excludes cardioembolic stroke. Fatal stroke based on superimposed clot. | Stroke only | Stroke only: 5.8% (15/259) |
| Gungor, 2018^18^ | Europe | Turkey |  | 4 | 262 | CTA or MRA TOF | NASCET criteria, stenosis >50% | modified TOAST criteria | Stroke only | Stroke only: 11.1% (29/262) |
| Hurford, 2020^19^ | Europe | UK |  | 4 | 1368 | CTA or MRA TOF | WASID>50% | modified TOAST criteria | Stroke and TIA | Stroke and TIA: 6.9% (94/1368); not reported separately |
| Logallo, 2014^20^ | Europe | Norway |  | 4 | 607 | MRI TOF, CTA, TCD | WASID, any stenosis; standardised TCD criteria | modified TOAST criteria | Stroke and TIA | Stroke and TIA 7.4% (45/607); not reported separately |
| Tsivgoulis, 2014^21^ | Europe | Greece |  | 3 | 467 | TCD screening, followed by MRI TOF | Standardised TCD criteria, MRI TOF criteria not specified | modified TOAST criteria | Stroke and TIA | Stroke and TIA 9.2% (43/467); not reported separately |
| Weber, 2010^22^ | Europe | Germany |  | 4 | 13584 | MRI TOF | WASID, >50% | modified TOAST criteria | Stroke and TIA | Stroke and TIA: 2.2% (304/13,584); not reported separately |
| Baracchini, 2016^23^ | Europe | Italy |  | 4 | 1134 | Screening with TCD, and then MRA TOF or CTA | WASID, stenosis >50% | Excludes cardioembolic stroke | Stroke only | Stroke: 8.7% (99/1134) |
| Weimar, 2006^24^ | Europe | Germany |  | 3 | 4157 | TCD, MRI TOF, CTA, DSA | WASID, >50% | modified TOAST criteria | Stroke only | Stroke: 6.5% (272/4157) |
| Weitzel-Mudersbach, 2012^25^ | Europe | Denmark |  | 3 | 195 | TCD | Standardised TCD criteria | All same-vessel stroke | TIA only | TIA: 8.2% (16/195) |
| Wityk, 1996^26^ | North America | United States |  | 3 | 274 | MRI TOF or TCD | >50% stenosis, measurement criteria not specified; standardised TCD criteria | modified TOAST criteria | Stroke or TIA | Stroke or TIA 9% (27/274); not reported separately |
| Lau, 2013^27^ | North America | United States |  | 5 | 697 | CTA | WASID, any stenosis | Modified TOAST criteria | Stroke only | Stroke: 25% (176/697) |
| Sacco, 1995^28^ | North America | United States |  | 4 | 438 | TCD | Standardised TCD criteria | Modified TOAST criteria | Stroke only | Total: 7.5% (33/438); -- White: 1.2% (1/82); Black: 6.5% (10/155); Hispanic: 10.7% (21/197) |
| Schaafsma, 2019^29^ | North America | Canada | Consecutive patients referred from the hospital stroke service between 2006 and 2014 for intracranial VW-MR imaging to clarify the etiology of an ischemic stroke or TIA. | 3 | 205 | CTA, DSA, MRI TOF, MRI VWI | CTA, DSA, MRI TOF criteria not reported; MRI VWI reported by panel of neuroradiologists | modified TOAST | Stroke and TIA | Stroke and TIA: 57% (116/205) with MRI VWI; using conventional imaging only (MRA TOF, CTA, DSA): 23% (48/205); stroke and TIA not reported separately |
| White, 2005^30^ | North America | United States | To compare subtype-specific ischaemic stroke incidence rates amongst Whites, Blacks and Hispanics in Northern Manhattan | 3 | 714 | MRI TOF, CTA, TCD | Not reported | modified TOAST | Stroke only | Stroke due to ICAD incidence rates per 100,000: Whites 3.4% (3/88); Hispanic 8.7% (13/149); Blacks 7.8% (15/191) |
| Koch, 2005^31^ | North America | United States | Stroke subtypes in Haitian-born patients in Miami | 3 | 126 | MRI TOF, DSA | Any stenosis, methods not reported | modified TOAST | Stroke only | 9% (11/126) |
| Lange, 2018^21^ | South America | Brazil |  | 4 | 359 | MRI TOF, CTA or DSA | WASID, >50% | Modified TOAST criteria | Stroke only | Stroke: 5% (18/359) |
| Bang, 2005^32^ | Asia | Korea |  | 4 | 497 | MRI TOF | WASID, >50% | Modified TOAST criteria | Stroke only | 24.3% (121/497) |

| Table 2: Recurrent Stroke in ICAD | | | | | | | | | | | | | |
| --- | --- | --- | --- | --- | --- | --- | --- | --- | --- | --- | --- | --- | --- |
| **Source** | **Region** | **Country** | **Modified Newcastle-Ottawa Scale Score (out of 9)** | **Patient numbers** | **Patient population** | **How ICAD was identified** | **ICAD definition** | **Symptomatic ICAD definition** | **Medical therapy** | **Recurrent cerebrovascular event definition** | **Follow-up period** | **Recurrent same-territory cerebrovascular event rate** | **Recurrent cerebrovascular event – any** |
| Saraf, 2020^33^ | Multi-regional | India and United States | 6 | 329 United States 35.6% (117/329);India 64.4% (212/329) |  | DSA, CTA, MRI TOF | WASID, >50% | Stroke or TIA same-territory as ICAD. Other potential causes not excluded. | At discretion of treating team; 70.2% on dual antiplatelet therapy, 95% on statin | Stroke only | 3 months | NA | Total: 18% (60/329); India: Recurrent stroke + TIA at 3 months: 8.5% (10/117); Recurrent stroke at 3 months: 1.7% (2/117); Recurrent TIA at 3 months: 6.8% (8/117) - United States: Recurrent stroke + TIA at 3 months: 23.6% (50/212); Recurrent stroke at 3 months: 21.7% (46/212); Recurrent TIA at 3 months: 1.9% (4/212) |
| Massot, 2014^34^ | Europe | Spain | 7 | 73 |  | Screening on TCD, confirmed on MRA or CTA | Standardised TCD criteria, MRI TOF and CTA criteria not reported | Stroke or TIA | Not reported | Stroke or TIA; | Median 22.4 +/- 7.6 months | NA | Total: 17.8% (13/73), Stroke: 13.7% (10/73), TIA: 6.8% (5/73) |
| Hoh, 2016^35^ | North America | United States | 5 | 188 |  | Not reported | WASID>50% | Stroke or TIA | At discretion of treating team, details not specified | Stroke and TIA | 12 month follow-up | NA | Total 9% (17/188); stroke 3.7% (7/188); TIA 5.3% (10/188) |
| Samaniego, 2009^36^ | North America | United States | 6 | 58 | .Medical arm of non-randomised study comparing intracranial stenting to medical therapy. | MRA TOF, DSA, TCD | Not reported | Stroke and TIA | Antithrombotic therapy, details not specified | Stroke and TIA | Mean 14 months | NA | Total 14% (8/58); Stroke: 5.1% (3/58); TIA: 8.6% (5/58) |
| Liu, 2015^37^ | Asia | China | 7 | 481 | Patients from Clopidogrel in High-Risk Patients with Acute Non-disabling Cerebrovascular Events (CHANCE) trial | MRI TOF | WASID, >50% | Not reported | Patients randomised to clopidogrel and aspirin vs aspirin only | Stroke only | Patients followed-up for 90 days, 0.7% loss to follow-up | NA | Stroke: 12.5% (60/481) |
| Mazighi, 2006^17^ | Europe | France | 7 | 102 | TIA or minor (nondisabling) ischaemic stroke | Screening on TCD, confirmed on MRI TOF, CTA or DSA | Standardized TCD criteria, DSA, MRI TOF and CTA criteria not specified | modified TOAST criteria | At discretion of treating team; dual antiplatelet 9%, single antiplatelet 38%, antiicoagulant 53% | Stroke or TIA | Mean follow-up 23.4 months | Stroke and TIA: 38.2% (39/102); Stroke: 13.7% (14/102); TIA: 24.5% (25/102) | Stroke and TIA: 40.2% (41/102); Stroke: 14.7% (15/102); TIA: 25.4% (26/102) |
| Wong, 2000^38^ | Asia | China | 6 | 705 |  | TCD | Standardised TCD criteria | Stroke and TIA | At discretion of treating team; 85% (599/705) on antithrombotic therapy | Stroke and TIA | 6 months | NA | Recurrent stroke, TIA, acute coronary syndrome or death: 18.2% (47/258) |
| Gouveia, 2014^39^ | Europe | Portugal | 5 | 158 | Patients presenting wih stroke or TIA who had TCD | TCD, CTA if TCD not possible | Standardised TCD criteria | Stroke or TIA in ICAD-affected vessel, excluding cardioembolic causes | Not reported | Stroke and TIA | Mean follow-up 13.63 months (SD 7.34) | NA | Recurrent stroke or TIA, 19% (14/72) |
| Fu, 2010^40^ | Asia | China | 8 | 293 |  | MRI TOF | WASID>50% | Stroke in territory of ICAD-affected vessel, excluding cardioembolic stroke | Not reported | Stroke only | Mean follow-up 22.9 months, SD 11.6 | NA | Recurrent stroke (haemorrhagic and ischaemic) 20.8% (61/293), recurrent ischaemic stroke 20.1% (59/293) |
| Romano, 2021^41^ | North America | United States | 8 | 105 |  | DSA, MRI TOF, CTA | WASID, >50% | modified TOAST criteria | Aggressive medical therapy based on the Stenosis (SAMMPRIS) trial medical regimen | Stroke or TIA in symptomatic territory; new infarcts in the territory on MRI at 6-8 weeks | 253 ± 31 days (mean ± SD), 2.9% (3/105) lost to follow-up | Stroke: 8.8% (2.7/100 patient-years); TIA: 5.9% (8.5/100 patient-years); Infarct on MRI B at 6-8 weeks : 24.7% (22/89) | NA |
| Zaidat, 2015^42^ | North America | United States | 5 | 53 | Medical arm of stenting trial the Vitesse Intracranial Stent Study for Ischemic Stroke Therapy (VISSIT) | CTA or MRI TOF | WASID, >70% | Any same-vessel stroke | Clopidogrel 75mg daily for first 3 months and aspirin (81-325mg daily) for the study duration. Statin (20-80mg daily). Antihypertensive if required | Stroke or TIA | 12 months | Stroke or TIA: 9.4% (5/53); Stroke: 5.7% (3/53) | NA |
| Lan, 2017^43^ | Asia | China | 7 | 95 |  | CT angiogram | WASID, >50% | Stroke only. Excluded cardioembolic causes. | Standard medical care, not otherwise specified | Stroke or TIA | 2 year follow-up, no loss to follow-up | Stroke and TIA: 21/95 (22.1%); stroke 11/95 (11.9%); TIA 10.5% 10/95 | NA |
| Leng, 2019^44^ | Asia | China | 8 | 245 |  | MRI TOF, CTA | WASID, >50% | Stroke in ICAD-affected vessel, excludes cardioembolic causes | At discretion on managing doctors. On discharge - 95.9% on antiplatelets (33.9% on dual antiplatelets), 88.2% on statins, 53.9% on antihypertensives. | Stroke or TIA, | 12 month follow-up, 1.6% (4/245) lost to follow-up | Stroke and TIA: 13.5% (33/245); stroke only: 8.2% (20/45); TIA only 5.3% (13/245) | NA |
| Fischer, 2020^45^ | Europe | Switzerland | 7 | 33 |  | TCD for screening, CTA or MRA to confirm | NASCET, any stenosis | Stroke or TIA in territory of ICAD-affected vessel. | At discretion of treating team. | Stroke and TIA | 3 years | Recurrent same-territory stroke or TIA 31.3% (10/33); not reported separately | NA |
| Feng, 2019^46^ | Asia | China | 6 | 153 |  | MRI TOF, CTA | WASID>50% | Stroke only | At discretion of treating team; all patients on antiplatelet therapy if not contraindicated | Stroke and TIA | 12 month follow-up. No loss to follow-up. | Recurrent same territory stroke or TIA 17.2% (26/153); Recurrent same-territory stroke 17/122 (13.9%) | NA |
| Kang, 2007^47^ | Asia | Korea | 5 | 133 |  | MRI TOF | Measurement technique not specified, any stenosis | Stroke in ICAD-affected vessel | Not specified | New diffusion restricting lesion within 1 week of initial stroke | 1 week | 50.9% (28/55) | NA |
| Yaghi, 2019^48^ | North America | United States | 2 | 25 |  | CTA | WASID, >70% | Stroke only, anterior circulation only | At discretion of treating team, details not specified | Stroke only | 90 days | 32.0% (8/25) | NA |
| Kim, 2016^49^ | Asia | Korea | 8 | 138 | Symptomatic intracranial atherosclerosis with stroke or TIA | MRI TOF followed by HR MRI with vessel wall imaging | WASID, >50% | Modified TOAST criteria | At discretion of managing doctors (67.4% on dual antiplatelet therapy, 79% on statin) | Stroke only | Median 18 month follow-up (IQR 11-27 months) | 28.3% (39/138) | NA |
| Shi, 2021^50^ | Asia | China | 7 | 59 |  | MRI VWI | WASID, >30% | Modified TOAST criteria | Not reported | Stroke or TIA | Mean follow-up 6.2 months (SD±4.1) | 20.7% (12/59) | NA |
| Sangha, 2017^51^ | North America | United States | 5 | 99 |  | CTA, DSA, MRA | WASID, >50% | modified TOAST criteria | Aggressive medical therapy (DAPT+statin) in 20.4%, high dose-statin only in 21.5%, DAPT only in 22.4% | Stroke only | 30 days, no loss to follow-up | 20.2% (20/99) | NA |
| Song, 2020^52^ | Asia | China | 5 | 60 |  | MRI TOF | WASID, >30% | modified TOAST criteria | Not reported | Stroke only | Median 12 months (range 1-18). No loss to follow-up. | 20% (12/60) | NA |
| Ssi-Yan-Kai, 2013^53^ | Europe | France | 4 | 16 |  | MRI TOF | WASID, >50% | Same-territory TIA | At discretion of medical team, details not specified | Stroke or TIA | 7 days. No loss to follow-up reported. | 18% (3/16) | NA |
| Kate, 2014^54^ | Asia | India | 6 | 45 |  | DSA, if not possible CTA, if not possible MRI TOF | WASID, any percent stenosis | modified TOAST criteria | Standard medical care, not otherwise specified | Stroke only | 90 day follow-up | 11.1% (5/45) | NA |
| Amin-Hanjani, 2016^55^ | North America | United States | 6 | 72 |  | MRI TOF | WASID criteria >50% | Stroke or TIA, circulation only | At discretion of treating team | Stroke or TIA | Median follow-up 23 months (IQR 14-25) | 13.50% | NA |
| Hurford, 2020^19^ | Europe | UK | 8 | 94 |  | MRI TOF, CTA if MRI comntraindicated, Doppler if CTA also contraindicated | WASID>50% | Stroke or TIA in ICAD-affected vessel | Dual antiplatelets for 1 month, with single antiplatelet thereafter; highdose statin therapy; treatment of hypertension with target of <130/80 | Stroke and TIA | Median follow-up 2.8 years (IQR 1·5–4·6) | Recurrent same-territory stroke or TIA: cumulative risk 12% (11/94); recurrent same-territory stroke cumulative risk 5.5% (5/94); recurrent same-territory TIA cumulative risk 7.6% (7/94) | Any stroke (ischaemic or haemorrhagic) or death cumulative risk: 23.4% (21/94) |
| Chimowitz, 2005^56^ | North America | United states | 8 | 569 | Patients enrolled in Comparison of Warfarin and Aspirin for Symptomatic Intracranial Arterial Stenosis (WASID) trial | DSA | WASID criteria >50% | modified TOAST criteria | Aspirin or warfarin | Stroke only | Mean follow-up 1.8 years, 2.3% (13/569) lost to follow-up | Aspirin group 15% (42/280), warfarin group 12.1% (35/289) | Any ischaemic stroke: aspirin group 20.4% (57/280); warfarin group 17% (49/289) |
| Liu, 2020^57^ | Asia | China | 8 | 930 | Patients from China Intracranial Atherosclerosis (CICAS) study | MRA TOF | WASID>50% | modified TOAST criteria | At discretion of treating team; dual antiplatelet therapy 12.6%; antithrombotic therapy 98%; statin 78% | Stroke only | 12 month follow-up | NA | 9.4% (87/930) |
| Shin, 2005^58^ | Asia | Korea | 7 | 200 |  | DSA or MRI | Not reported | modified TOAST criteria. | Not reported | Stroke only | Mean follow-up 2 years (range 7 months to 3.5 years) | 8% (16/200) | 9% (18/200) |
| Weimar, 2006^24^ | Europe | Germany | 5 | 132 |  | TCD, MRI TOF, CTA, DSA | WASID, >50% | Stroke and TIA, modified TOAST criteria | At discretion of medical team, details not reported | Stroke only | 12 months | NA | 6.1% (8/132) |
| Thijs, 2000^59^ | North America | United States | 4 | 52 | Stroke or TIA | MRI TOF, CTA, TCD | NASCET, >50% | Stroke or TIA, modified TOAST criteria | At discretion of treating medical team; warfarin 54% (28/52), antiplatelets 37% (19/52), 8% (4/52) | Stroke or TIA | Mean follow up period 16.7 months | NA | 55.8% (29/52) |
| Lange, 2018^60^ | South America | Brazil | 6 | 18 |  | MRI TOF, CTA or TCD; DSA performed if contradiction between scans; repeat scan performed to confirm persistence of stenosis | WASID, >50% |  | At discretion of treating team; (83.3% on antiplatelet therapy) | Stroke only | Mean follow-up 21.6±15.1 months | NA | 38.9% (7/18) |
| Wong, 2003^61^ | Asia | China | 6 | 258 |  | TCD | Standardised TCD criteria | Stroke and TIA | At discretion of treating team | Stroke and TIA | 42 months (mean 28 +/- 5 months) | NA | 25.5% (66/258) |
| Vaishnav, 2018^62^ | Asia | India | 4 | 87 |  | MRI TOF, DSA | WASID, >50% | modified TOAST criteria | At discretion of treating team, details not specified | Stroke only | 12 months. Loss to follow-up not reported. | NA | 2.3% (2/87) |
| Tang, 2011^63^ | Asia | China | 6 | 61 | Medical arm of stenting vs medical treatment for severe symptomatic ICAD | DSA | WASID, >70% | Stroke only | At discretion of treating team, details not specified | Stroke or TIA | 3-36 months (mean 17.3 months) | NA | 19.7% (12/61) (11 stroke, 1 TIA) |
| Ram, 2017^64^ | Asia | India | 6 | 100 | Consecutive patients of ischaemic stroke due to ICAS, admitted to Nizam's Institute of Medical Sciences, Telangana, with informed consent obtained | MRI TOF | WASID, >50% | Not reported | At discretion of treating stroke team. Majority of patients on dual antiplatelet therapy, on statin and vascular risk factors controlled | Stroke | Stroke only, follow-up at 1 year. Loss to follow-up not reported. | 16% (16/100) | 17% (17/100) |
| Uehara, 2018^65^ | Asia | Japan | 5 | 82 | Patients with TIA (defined as <24 hrs of symptoms regardless of imaging findings) caused by intracranial arterial occlusive lesions who were admitted to the stroke care unit within 48 h of the onset of a TIA between April 2008 and November 2015 | MRI TOF or DSA | WASID, >50% | TIA only | Not reported | Stroke only | 90 days, loss to follow-up not reported | NA | 14.6% (12/82) within 90 days |
| Leung, 2015^66^ | Asia | China | 8 | 49 | First ever acute ischaemic stroke attirbuted to high grade stenosis, antithrombotic naïve | DSA | WASID, >70% | Stroke in ICAD-affected vessel, excluded cardioembolic or extracranial carotid atherosclerosis | Dual antiplatelets prescribed for 4 weeks, followed by aspirin alone. All patients received statin treatment. Aggressive conntrol of other risk factors. | Stroke only | 12 month follow-up, loss to follow-up not reported | 12.3% (6/49) | 14.3% (7/49) |
| Weber, 2010^22^ | Europe | Germany | 5 | 197 | Consecutive patients with acute stroke or transient ishaemic attack (TIA) due to symptomatic 50-99% intracranial atherosclerotic stenoses (ICAS), prospectively recruited from 19 German stroke centres | MRI TOF | WASID, >50% | Stroke and TIA, modified TOAST criteria | At discretion of treating team. Antiplatelets 165/197 (83.8%); anticoagulation 20/197 (10.2%); statin 97/197 (49.2%); antihypertensives 54% 107/197 | Stroke only | Mean follow-up time was 23.7 months. | NA | 12 months: 17.9%; 3 years: 23.3% |
| Sanchez-Tapia, 2020^67^ | Asia | Philippines | 5 | 109 |  | MRI TOF, CTA, TCD | WASID, any stenosis; standardised TCD criteria | Stroke only, modified TOAST criteria | Not reported | Stroke only | 12 months, loss to follow-up not reported | NA | 1.8% (2/109) |
| Derdeyn, 2014^68^ | North America | United States | 9 | 227 | Patients enrolled in SAMMPRIS trial medical arm | DSA | WASID>70% | Stroke or TIA in territory of ICAD-affected vessel, excluding patients with cardioembolic or carotid sources | Aspirin for the duration of follow-up, clopidogrel for 90 days after enrolment. Intensive management of blood pressure, LDL and other risk factors. | Stroke only | 2 year follow-up period; 11% (24/227) lost to follow-up | 34/227 (15%) | Any recurrent stroke or death 23% (51/227) |
| Zhou 2014^69^ | Asia | China | 6 | 120 |  | CTA | WASID >50% | Any stroke | Atorvastatin 10, 20 or 40mg; antiplatelets not reported | Stroke or TIA | 52 week follow-up | NA | Any stroke or TIA  17/120 (14%) |
| Arenillas 2003^70^ | Europe | Spain | 5 | 71 |  | TCD | Standardised TCD criteria | Same-territory stroke without any other potential causes | Not reported | Stroke or TIA | 1 year | 4.2% (3/71) | 9.9% (7/71) |

| Table 3: Prevalence of ICAD in the general population | | | | | | | | | |
| --- | --- | --- | --- | --- | --- | --- | --- | --- | --- |
| Source | Region | Country | Modified Newcastle-Ottawa Scale Score (total; of 5) | Population screened | Mean age | Patient numbers | How ICAD was identified | ICAD definition | Prevalence of ICAD |
| Suemoto, 2018^71^ | South America | Brazil | 5 | Subjects dying from natural death of unknown causes who underwent autopsy | 71.3±11.7 years | 661 | Histopathology | >50% stenosis, other criteria not specified | 58.5% (387/661) |
| Qiao, 2017^72^ | North America | United States | 5 | Black and white participants, recruited in middle age (45-64) | 77.6 (SD) +/- 5.3 years | 1752 | MRI black blood vessel wall imaging | WASID, >1% | 36.4% (637/1752); Black: 36.3% (188/518) ; White: 32.4% (400/1234) |
| Ahn, 2018^73^ | Asia | Korea | 4 | Kangbuk Samsung Health Study | 37.4 | 17,172 | TCD | Standardised TCD criteria | 3.55% |
| Chen, 2019^74^ | Asia | China | 4 | Individuals over 40, registered households in Dehui City, Jilin province | Not reported | 3654 | TCD | Standardised TCD criteria | 13.70% |
| Jin, 2017^75^ | Asia | China | 4 | Asymptomatic subjects aged >30 years sfrom 13 villages in Shanhe County, Hebei Province; | 55.3 | 2589 | TCD | Standardised TCD criteria | 4.7% (122/2589) |
| Kamal, 2014^76^ | Asia | India | 4 | Adults who underwent MRI brain for indications other than stroke or TIA | 37.1 | 200 | MRI TOF | WASID criteria, any stenosis | 34.5% (69/200) |
| Kang, 2014^77^ | Asia | Korea | 4 | Stroke-free patients who consecutively visited a general health promotion center | 52 | 361 | MRI TOF | WASID >50% stenosis | 20.5% (74/361) |
| Matsui, 2016^78^ | Asia | Japan | 4 | Healthy Japanese volunteers with no history of stroke, mean age | 62.0 (SD) +/- 8.5 |  | MRI TOF | Uehara criteria | 5.9% (166/2807) |
| Nam, 2017^79^ | Asia | Korea | 4 | Consecutive healthy subjects who visited the Seoul National University Hospital Health Promotion Center | 56 | 3159 | MRI TOF | WASID, >50% | 2.6% (82/3159) |
| Park, 2006^80^ | Asia | Korea | 4 | Asymptomatic Korean adults who voluntarily visited a tertiary referral hospital for a health screening | 53 | 835 | MRI TOF | WASID, >50% | 3.0% (25/835) |
| Song, 2020^81^ | Asia | China | 4 | General population from urban and rural areas | 63 (IQR) ± 13 years | 3640 | TCD | Standardised TCD criteria | 17.3% (629/3640) |
| Leung, 1993^82^ | Asia | China | 4 | Consecutive Chinese patients undergoing autopsy - 8.8% determined to have death from cerebrovascular disease from autopsy; | mean age not reported, >50% over 50 | 114 | Histopathology | >50% stenosis, other criteria not specified | Prevalence of ICAD amongst adults 31.4% (36/114) |
| Sun, 2020^83^ | Asia | China | 4 | Subjects aged 40+ years who were stroke free and living in Kongcun Town, Shandong | 57.6 (SD) ±10.4 | 2019 | TCD | Standardised TCD criteria | 7.6% (153/2019) |
| Wang, 2014^84^ | Asia | China | 4 | Subpopulation of Kailuan study group (employees and retirees of a coal mine company), aged 40+ without vascular disease | 55.2 (SD)± 11.8 | 5393 | TCD | Standardised TCD criteria | 13.2% (713/5393) |
| Wong, 2007^85^ | Asia | China | 4 | Asymptomatic villagers aged >=40 years in Liangbei County in central rural China | 53.5 years (SD) ±10.7 years | 590 | TCD | Standardised TCD criteria | 6.9% (41/590) |
| Zhai, 2018^86^ | Asia | China | 4 | All inhabitants aged 35 years and older, living in 5 villages of Shunyi, a suburb district of Beijing were invited; | 57.2 years (SD)±9.4 | 1237 | MRI TOF | WASID, any stenosis | 15.7% (194/1237) |
| Shitara, 2019^87^ | Asia | Japan | 3 | General population free from stroke and coronary heart disease, myocardial infarction or revascularisation | 68.0 years | 709 | MRI TOF | Criteria not specified | 25.2% (208/709) |
| Gutierrez, 2015^88^ | North America | United States | 2 | Subjects drawn from the Brain Arterial Remodelling Study (BARS) | 55(SD)±17 | 196 | Histopathology | Presence of pathological intima thickening, fibrous cap atheroma, thin fibrous cap atheroma or intima thickening excluding xanthomas or minor degrees of adaptive intima thickening | 46% |

| Table 4: Prevalence of ICAD amongst stroke patients | | | | | | | | |
| --- | --- | --- | --- | --- | --- | --- | --- | --- |
| **Source** | **Region** | **Country** | **Modified Newcastle-Ottawa Scale Score (out of 5)** | **Patients numbers** | **Patient population details** | **How ICAD was identified** | **ICAD definition** | **Prevalence of ICAD in high-risk group** |
| Mazighi, 2008^89^ | Europe | France | 5 | 259 | Fatal stroke | Histopathology | Raymond Escourolle criteria | 62.2% (161/259) |
| Zhou, 2020^90^ | Asia | China | 5 | 1101 | Patients with first ischaemic stroke. Patients either Han Chinese or Zhuang Chinese. | CTA | WASID, any stenosis | Overall: 63.6% Han Chinese: 64.5% (483/749) Zhuang Chinese 61.6% (217/352). |
| Ovesen, 2013^91^ | Europe | Denmark | 5 | 652 | Stroke and TIA patients | CTA | WASID, >30% | 15.5% (101/652) |
| Zhang, 2019^92^ | Asia | China | 5 | 207 | Stroke patients | CTA | WASID, >50% | 64.7% (134/207) |
| Ratanakorn, 2012^93^ | Asia | Thailand | 4 | 756 | Stroke and TIA patients | TCD | Standardised TCD criteria | 52.6% (398/756) |
| Barreto-Neto, 2016^94^ | South America | Brazil | 4 | 106 | Stroke patients | CTA | Not specified | Mild: 50% (19/106); Moderate: 18% (7/106); Severe: 32% (12/106) |
| Lee, 2015^95^ | Asia | Korea | 4 | 223 | Stroke patients with possible cardioembolic source | MRI TOF | >50% signal loss of MRA TOF | 32.3% (72/223) |
| Palacio, 2014^96^ | Multi-regional | US, Canada, Spain, Mexico, Ecuador, Peru, Chile, Argentina | 4 | 2908 | Stroke patients | MRI TOF, CTA | Not reported | 17.3% (503/2908) |
| Leng, 2021^97^ | Asia | China, UK | 4 | Total: 1969, UK: 1278, Hong Kong: 691 | Stroke and TIA patients | MRI TOF, CTA, doppler | WASID, >50% | Total: 28% (554/1969), UK 20% (257/1278), Hong Kong 43% (297/691) |
| Kim, 2010^4^ | Asia | Korea | 4 | 1012 | Stroke patients | MRI TOF | WASID, >50% | 26.9% (272/1012) |
| Hua, 2019^98^ | Asia | China | 4 | 9346 | Stroke patients | TCD screening, then MRA TOF or CTA | WASID, >50% | 30.8% (2878/9346) |
| Kim, 2013^99^ | Asia | Korea | 4 | 1043 | First-ever stroke patients | MRI TOF | WASID, >50% | 51.2% (534/1043) |
| Guo, 2018^100^ | Asia | China | 4 | 1116 | Non-disabling stroke patients | CTA, MRA, DSA | Samuel's method for CTA or MRA, Barnett's method on DSA (reference) | Total 27.9% (311/1116); moderate 13.1% (146/1116); severe 11.4% (127/1116; artery occlusion 3.4% (38/1116) |
| Park, 2017^101^ | Asia | Korea | 4 | 9506 | Minor stroke and high-risk TIA patients | MRI TOF | Not reported | 35.6% (3387/9506) |
| Park, 2021^102^ | Asia | Korea | 4 | 2670 | Stroke patients with AF | MRI TOF | WASID, >50% | 24.8% (661/2670) |
| Park, 2015^103^ | Asia | Korea | 4 | 679 | Stroke patients | MRI TOF | WASID, >50% | 46.8% (318/679) |
| Park, 2007^104^ | Asia | Korea | 4 | 378 | Stroke patients | MRI TOF | Uehara, 1994 criteria | 42.1% (159/378) |
| Weber, 2010^22^ | Europe | Germany | 4 | 13584 | Stroke and TIA patients | MRI TOF | WASID, >50% | 5.4% (736/13,584) |
| Li, 2003^105^ | Asia | China | 4 | 699 | Stroke patients | TCD | Standardised TCD criteria | 49.4% (345/699) |
| Uchiyama, 2019^106^ | Europe, Asia, Australia and North America | 21 countries | 3 | 3628 | Minor stroke and TIA patients | MRI TOF, CTA | Percent stenosis >50%, criteria not specified | 19.6% (67/341) |
| Zhang, 2018^107^ | Asia | China | 3 | 226 | Non-traumatic intracranial haemorrhage patients | CTA | WASID, any stenosis | 25.2% (57/226) |
| Li, 2003^105^ | Asia | China | 3 | 84 | Stroke patients with extracranial carotid atherosclerosis | TCD | Standardised TCD criteria | 84.5% (71/84) |
| Kamtchum-Tatuene, 2020^108^ | Africa | Malawi | 3 | 26 | Stroke patients | TCD | Standardised TCD criteria | 19.2% (5/26) |
| De Silva, 2009^109^ | Asia | Singapore | 3 | 183 | Ethnic-Chinese stroke patients | TCD, if inadequate imaging then MRI TOF | Standardised TCD criteria | 57% (77/135) |
| Viaro, 2012^110^ | Europe | Italy | 3 | 292 | Stroke and TIA patients | TCD | Standardised TCD criteria | 20.2% (59/292) |
| Von Sarnowski^111^ | Europe | Based in Germany, recruitment from 15 European countries | 3 | 1612 | Stroke and TIA patients aged 18-55 | TCD | Standardised TCD criteria | 11.8% (190/1612) |
| Li, 2015^112^ | Asia | China | 3 | 412 | Stroke patients | CTA | WASID, any stenosis | 51.2% (211/412) |
| Tawfik, 2009^113^ | Africa | Egypt | 3 | 41 | First-ever stroke patients | TCD | Standardised TCD criteria | 9.8% (4/41) |
| Wang, 2019^114^ | Asia | China | 2 | 4012 | Stroke patients and controls | MRI TOF | WASID, >50% | Non-stroke: 316/1434 (22.0%); Stroke: 1026/2578 (38.8%) |
| Markus, 2007^115^ | Europe | UK | 4 | 215 | Stroke patients | MRI TOF, CTA, TCD | modified TOAST criteria | Total ICAD: 12% (26/215); amongst black patients: 17.9% (24/134); amongst white patients: 2.5% (2/81) |
| Moussouttas, 2006^116^ | North America | United States | 3 | 205 | Stroke and TIA patients | TCD, CTA, MRA | Not reported | South Asian: 20% (20/99) European-American: 7% (7/106) |
| Meseguer, 2010^117^ | Europe | France | 4 | 1823 | TIA patients | TCD | Standardised TCD criteria, stenosis >50% | 8.8% (161/1823) |
| Shi, 2014^118^ | Asia | China | 4 | 351 | Stroke patients aged <55 | MRI TOF, CTA, DSA | WASID >50% | 34.5% (121/351) |
| Kim 2010^119^ | Asia | Korea | 4 | 1955 | All stroke patients | MRI TOF | WASID, >50% | 26.4% (516/1955) |

| Table 5: Prevalence of ICAD in high-risk groups | | | | | | | | |
| --- | --- | --- | --- | --- | --- | --- | --- | --- |
| **Source** | **Region** | **Country** | **Modified Newcastle-Ottawa Scale Score (out of 5)** | **Patients numbers** | **High-risk group definition** | **How ICAD was identified** | **ICAD definition** | **Prevalence of ICAD in high-risk group** |
| Hoshi, 2007^120^ | Asia | Japan | 4 | 226 | Stroke-free patients refered for primary prevention of stroke. Mean age 67. | MRI TOF | Methods described by Uehara et al (reference) | 20.4% (46/226) |
| Suri, 2016^121^ | North America | United States | 4 | 1765 | Patients participating in the Atherosclerosis Risk in Communities (ARIC) study. Mean age 76 ± 5.3 years | MRI TOF | WASID, >50% stenosis | 10.6% (188/1765); |
| Bae, 2007^122^ | Asia | Korea | 4 | 1208 | Presence of vascular risk factors, age >40. Mean age 56.9 (IQR 48-65), | TCD | Standardised TCD criteria | 296/1208 (24.5%) |
| Park, 2013^123^ | Asia | Korea | 4 | 682 | Stroke-free patients refered for primary prevention of stroke. Mean age 64±10 years. | MRI TOF | WASID, >50% | 8.8% (60/682) |
| Liu, 2020^124^ | Asia | China | 4 | 1062 | Patients with symptomatic coronary disease | CTA | WASID, >50% stenosis | 23.1% (245/1062) |
| Jellinger, 2012^125^ | Europe | Germany | 4 | 100 | Patients with severe systemic atherosclerosis | Histopathology | Macroscopic assessment, histological examination of multiple paraffin-embedded blocks | 54% (54/100) |
| Alkan, 2009^126^ | Europe | Turkey | 4 | 183 | Patients with extracranial carotid disease planned for coronary artery bypass grafts | DSA | Samuels, 2000 criteria | Any ICAD 27.9% (51/183); ICAD >50% 16.4% (30/183) |
| Li, 2017^127^ | Asia | China | 4 | 5209 | Patients with chronic kidney disease | TCD | Standardised TCD criteria | 13.1% (684/5209) |
| Zwartbol, 2019^128^ | Europe | Netherlands | 4 | 130 | History of vascular disease (cerebrovascular disease, peripheral arterial disease, coronary artery disease, or abdominal aortic aneurysm) | MRI VWI | Criteria defined by Lindenholz et al | 96% (125/130) |
| Lopez-Cancio, 2011^129^ | Europe | Spain | 4 | 933 | >50 years with a moderate-high vascular risk | MRI TOF, TCD | Standardised TCD criteria, MRI TOF criteria not reported | 8.6% (80/933) |
| Kim, 2011^130^ | Asia | Korea | 4 | 935 | Stroke-free patients refered for primary prevention of stroke. | TCD | Standardised TCD criteria | 19.7% (184/935) |
| Modesti, 2017^131^ | Europe | Italy | 3 | 96 | Chinese patients with newly diagnosed T2DM. | TCD | Standardized TCD criteria | Chinese with T2DM: 17.7% (17/96) |
| Abd-Allah et al, 2018^132^ | Africa | Egypt | 3 | 153 | Presence of vascular risk factors | TCD | Standardised TCD criteria | 13.1% (20/153) |
| Abd-Allah et al, 2014^133^ | Africa | Egypt | 3 | 118 | Presence of coronary artery disease | TCD | Standardised TCD criteria | 11.9% (14/118) |
| Lee, 2003^134^ | Asia | Korea | 3 | 142 | Patients steno-occlusive extracranial carotid disease, >30% | DSA | WASID, >30% | 56.3% (80/142) |
| Oh, 2015^135^ | Asia | Korea | 3 | 1,827 | Patients with coronary artery calcification on CT scan | TCD | Standardised TCD criteria | 4.4% (80/1827) |
| Valaikiene, 2019^136^ | Europe | Lithuania | 3 | 346 | 3 vessel and/or left stem coronary artery disease | TCD | Standardised TCD criteria | 63.6% (220/346) |
| Elmore, 2003^137^ | North America | United States | 2 | 510 | Patients referred for the evaluation of asymptomatic carotid bruits | TCD | Standardised TCD criteria | 12.9% (66/510) |
| Salem, 2010^138^ | Middle East | Egypt | 2 | 40 | Age above 40 years and presenting with or with a past history of coronary ischaemic heart disease | MRI TOF | Not reported | 52.5% (21/40) |
| Elwan, 2014^139^ | Middle East | Egypt | 2 | 100 | Patients with type 2 diabetes | MRI TOF | WASID, >1% | 18% (18/100) |

1.	Wells GA, Shea B, O’Connell D, Peterson J, Welch V, Losos M, Tugwell P. The Newcastle-Ottawa Scale (NOS) for assessing the quality of nonrandomised studies in meta-analyses. 2000.

2.	Pavlin‐Premrl D, Waters M, Thijs V, Mitchell PJ, Yassi N, Parsons MW, Weir L, Pesavento L, Maingard J, Cloud G. Symptomatic Intracranial Atherosclerosis in an Australian Context: Common and Underdiagnosed in Multiple Ethnic Groups. *Stroke: Vascular and Interventional Neurology*. 2025;5:e001397.

3.	Chang KC, Chuang IC, Huang YC, Wu CY, Lin WC, Kuo YL, Lee TH, Ryu SJ. Risk factors outperform intracranial large artery stenosis predicting unfavorable outcomes in patients with stroke. *BMC Neurol*. 2019;19:180. doi: 10.1186/s12883-019-1408-1

4.	Kim BS, Jung HS, Bang OY, Chung CS, Lee KH, Kim GM. Elevated serum lipoprotein(a) as a potential predictor for combined intracranial and extracranial artery stenosis in patients with ischemic stroke. *Atherosclerosis*. 2010;212:682-688. doi: 10.1016/j.atherosclerosis.2010.07.007

5.	Li M, Huang Y, Lin H, Chen Y. Association of uric acid with stenosis of intracranial and extracranial arteries in elderly patients with cerebral infarction. *Neurol Sci*. 2019;40:957-961. doi: 10.1007/s10072-019-03737-2

6.	Park JH, Kim BS, Ovbiagele B. Concomitant diabetes or hypertension with metabolic syndrome on the extent of intracranial atherosclerotic stenosis. *Neurol Sci*. 2020;41:387-395. doi: 10.1007/s10072-019-04105-w

7.	Kaul S, Sunitha P, Suvarna A, Meena A, Uma M, Reddy J. Subtypes of ischemic stroke in a metropolitan city of South India (one year data from a hospital based stroke registry). 2002.

8.	Kim BS, Chung PW, Park KY, Won HH, Bang OY, Chung CS, Lee KH, Kim GM. Burden of Intracranial Atherosclerosis Is Associated With Long-Term Vascular Outcome in Patients With Ischemic Stroke. *Stroke*. 2017;48:2819-2826. doi: 10.1161/STROKEAHA.117.017806

9.	Lee TH, Hsu WC, Chen CJ, Chen ST. Etiologic study of young ischemic stroke in Taiwan. *Stroke*. 2002;33:1950-1955. doi: 10.1161/01.str.0000021409.16314.16

10.	Lei C, Wu B, Liu M, Chen Y. Risk factors and clinical outcomes associated with intracranial and extracranial atherosclerotic stenosis acute ischemic stroke. *J Stroke Cerebrovasc Dis*. 2014;23:1112-1117. doi: 10.1016/j.jstrokecerebrovasdis.2013.09.024

11.	Man BL, Fu YP, Chan YY, Lam W, Hui CF, Leung WH, Wong KS. Use of magnetic resonance angiography to predict long-term outcomes of ischemic stroke patients with concurrent stenoses in Hong Kong. *Cerebrovasc Dis*. 2009;28:112-118. doi: 10.1159/000223435

12.	Park JH, Kwon HM. Association between metabolic syndrome and previous ischemic lesions in patients with intracranial atherosclerotic stroke. *Clin Neurol Neurosurg*. 2008;110:215-221. doi: 10.1016/j.clineuro.2007.10.016

13.	Kaul S, Alladi S, Jabeen SA, Bandaru VCSS, Ankem U, Mekala S, Naik GS. Intracranial Atherosclerosis is the Most Common Stroke Subtype: Ten-year Data from Hyderabad Stroke Registry (India). *Ann Indian Acad Neurol*. 2018;21:209-213. doi: 10.4103/aian.AIAN_86_18

14.	Niu JW, Gao S, Cui LY, Peng B, Zhu YC, Ni J, Zhou LX, Yao M, Xu WH. Intracranial atherosclerosis in Chinese young adult stroke patients. *J Stroke Cerebrovasc Dis*. 2014;23:1519-1523. doi: 10.1016/j.jstrokecerebrovasdis.2013.12.030

15.	Wong KS, Huang YN, Gao S, Lam WW, Chan YL, Kay R. Intracranial stenosis in Chinese patients with acute stroke. *Neurology*. 1998;50:812-813. doi: 10.1212/wnl.50.3.812

16.	Homburg PJ, Plas GJ, Rozie S, van der Lugt A, Dippel DW. Prevalence and calcification of intracranial arterial stenotic lesions as assessed with multidetector computed tomography angiography. *Stroke*. 2011;42:1244-1250. doi: 10.1161/STROKEAHA.110.596254

17.	Mazighi M, Tanasescu R, Ducrocq X, Vicaut E, Bracard S, Houdart E, Woimant F. Prospective study of symptomatic atherothrombotic intracranial stenoses: the GESICA study. *Neurology*. 2006;66:1187-1191. doi: 10.1212/01.wnl.0000208404.94585.b2

18.	Gungor L, Polat M, Ozberk MB, Avci B, Abur U. Which Ischemic Stroke Subtype Is Associated with Hyperhomocysteinemia? *J Stroke Cerebrovasc Dis*. 2018;27:1921-1929. doi: 10.1016/j.jstrokecerebrovasdis.2018.02.033

19.	Hurford R, Wolters FJ, Li L, Lau KK, Küker W, Rothwell PM, Cohort OVSP. Prevalence, predictors, and prognosis of symptomatic intracranial stenosis in patients with transient ischaemic attack or minor stroke: a population-based cohort study. *Lancet Neurol*. 2020;19:413-421. doi: 10.1016/S1474-4422(20)30079-X

20.	Logallo N, Naess H, Waje-Andreassen U, Thomassen L. Prevalence of intracranial stenosis in a Norwegian ischemic stroke population. *J Stroke Cerebrovasc Dis*. 2014;23:1611-1615. doi: 10.1016/j.jstrokecerebrovasdis.2013.12.049

21.	Tsivgoulis G, Vadikolias K, Heliopoulos I, Katsibari C, Voumvourakis K, Tsakaldimi S, Boutati E, Vasdekis SN, Athanasiadis D, Al-Attas OS, et al. Prevalence of symptomatic intracranial atherosclerosis in Caucasians: a prospective, multicenter, transcranial Doppler study. *J Neuroimaging*. 2014;24:11-17. doi: 10.1111/j.1552-6569.2012.00707.x

22.	Weber R, Kraywinkel K, Diener HC, Weimar C, Collaboration GSS. Symptomatic intracranial atherosclerotic stenoses: prevalence and prognosis in patients with acute cerebral ischemia. *Cerebrovasc Dis*. 2010;30:188-193. doi: 10.1159/000317107

23.	Baracchini C, Anzola GP, Cenciarelli S, Diomedi M, Bella R, Tonon A, Braga M, Zedde ML, Zanferrari C, Del Sette M, et al. Italian symptomatic intracranial atherosclerosis study (ISIDE) : A multicenter transcranial ultrasound evaluation. *Neurol Sci*. 2016;37:1645-1651. doi: 10.1007/s10072-016-2642-6

24.	Weimar C, Goertler M, Harms L, Diener HC. Distribution and outcome of symptomatic stenoses and occlusions in patients with acute cerebral ischemia. *Arch Neurol*. 2006;63:1287-1291. doi: 10.1001/archneur.63.9.1287

25.	von Weitzel-Mudersbach P, Johnsen SP, Andersen G. Intra-and extracranial stenoses in TIA–Findings from the Aarhus TIA-study: A prospective population-based study. *Perspectives in Medicine*. 2012;1:207-210.

26.	Wityk RJ, Lehman D, Klag M, Coresh J, Ahn H, Litt B. Race and sex differences in the distribution of cerebral atherosclerosis. *Stroke*. 1996;27:1974-1980. doi: 10.1161/01.str.27.11.1974

27.	Lau AY, Wong KS, Lev M, Furie K, Smith W, Kim AS. Burden of intracranial steno-occlusive lesions on initial computed tomography angiography predicts poor outcome in patients with acute stroke. *Stroke*. 2013;44:1310-1316. doi: 10.1161/STROKEAHA.111.672741

28.	Sacco RL, Kargman DE, Gu Q, Zamanillo MC. Race-ethnicity and determinants of intracranial atherosclerotic cerebral infarction. The Northern Manhattan Stroke Study. *Stroke*. 1995;26:14-20. doi: 10.1161/01.str.26.1.14

29.	Schaafsma JD, Rawal S, Coutinho JM, Rasheedi J, Mikulis DJ, Jaigobin C, Silver FL, Mandell DM. Diagnostic Impact of Intracranial Vessel Wall MRI in 205 Patients with Ischemic Stroke or TIA. *AJNR Am J Neuroradiol*. 2019;40:1701-1706. doi: 10.3174/ajnr.A6202

30.	White H, Boden-Albala B, Wang C, Elkind MS, Rundek T, Wright CB, Sacco RL. Ischemic stroke subtype incidence among whites, blacks, and Hispanics: the Northern Manhattan Study. *Circulation*. 2005;111:1327-1331. doi: 10.1161/01.CIR.0000157736.19739.D0

31.	Koch S, Pabon D, Rabinstein AA, Chirinos J, Romano JG, Forteza A. Stroke etiology among Haitians living in Miami. *Neuroepidemiology*. 2005;25:192-195. doi: 10.1159/000087449

32.	Bang OY, Lee MA, Lee JH, Kim JW, Lee PH, Joo IS, Huh K. Association of metabolic syndrome and C-reactive protein levels with intracranial atherosclerotic stroke. *Journal of Clinical Neurology*. 2005;1:69-75.

33.	Saraf U, Prabhakaran S, Arun K, Babiker A, Rajendran A, Kesavadas C, Sylaja PN. Comparison of Risk Factors, Treatment, and Outcome in Patients with Symptomatic Intracranial Atherosclerotic Disease in India and the United States. *Ann Indian Acad Neurol*. 2020;23:265-269. doi: 10.4103/aian.AIAN_549_19

34.	Massot A, Giralt D, Penalba A, Garcia-Berrocoso T, Navarro-Sobrino M, Arenillas JF, Ribó M, Molina CA, Alvarez-Sabín J, Montaner J, et al. Predictive value of ankle-brachial index and PAI-1 in symptomatic intracranial atherosclerotic disease recurrence. *Atherosclerosis*. 2014;233:186-189. doi: 10.1016/j.atherosclerosis.2013.12.034

35.	Hoh BL, Gong Y, McDonough CW, Waters MF, Royster AJ, Sheehan TO, Burkley B, Langaee TY, Mocco J, Zuckerman SL, et al. CYP2C19 and CES1 polymorphisms and efficacy of clopidogrel and aspirin dual antiplatelet therapy in patients with symptomatic intracranial atherosclerotic disease. *J Neurosurg*. 2016;124:1746-1751. doi: 10.3171/2015.6.JNS15795

36.	Samaniego EA, Hetzel S, Thirunarayanan S, Aagaard-Kienitz B, Turk AS, Levine R. Outcome of symptomatic intracranial atherosclerotic disease. *Stroke*. 2009;40:2983-2987. doi: 10.1161/STROKEAHA.109.549972

37.	Liu L, Wong KS, Leng X, Pu Y, Wang Y, Jing J, Zou X, Pan Y, Wang A, Meng X, et al. Dual antiplatelet therapy in stroke and ICAS: Subgroup analysis of CHANCE. *Neurology*. 2015;85:1154-1162. doi: 10.1212/WNL.0000000000001972

38.	Wong KS, Li H, Chan YL, Ahuja A, Lam WW, Wong A, Kay R. Use of transcranial Doppler ultrasound to predict outcome in patients with intracranial large-artery occlusive disease. *Stroke*. 2000;31:2641-2647. doi: 10.1161/01.str.31.11.2641

39.	Gouveia A, Sargento-Freitas J, Penetra J, Silva F, Machado C, Cordeiro G, Cunha L. Recurrence in intracranial atherosclerotic disease: a stenosis-based analysis. *J Stroke Cerebrovasc Dis*. 2014;23:2080-2084. doi: 10.1016/j.jstrokecerebrovasdis.2014.03.015

40.	Fu JH, Chen YK, Chen XY, Mok V, Wong KS. Coexisting small vessel disease predicts poor long-term outcome in stroke patients with intracranial large artery atherosclerosis. *Cerebrovasc Dis*. 2010;30:433-439. doi: 10.1159/000319574

41.	Romano JG, Prabhakaran S, Nizam A, Feldmann E, Sangha R, Cotsonis G, Campo-Bustillo I, Koch S, Rundek T, Chimowitz MI, et al. Infarct Recurrence in Intracranial Atherosclerosis: Results from the MyRIAD Study. *J Stroke Cerebrovasc Dis*. 2021;30:105504. doi: 10.1016/j.jstrokecerebrovasdis.2020.105504

42.	Zaidat OO, Fitzsimmons BF, Woodward BK, Wang Z, Killer-Oberpfalzer M, Wakhloo A, Gupta R, Kirshner H, Megerian JT, Lesko J, et al. Effect of a balloon-expandable intracranial stent vs medical therapy on risk of stroke in patients with symptomatic intracranial stenosis: the VISSIT randomized clinical trial. *JAMA*. 2015;313:1240-1248. doi: 10.1001/jama.2015.1693

43.	Lan L, Leng X, Ip V, Leung T, Soo Y, Abrigo J, Wong KS. Prolonged Perfusion Predicts Recurrent Ischemic Stroke but not Transient Ischemic Attack in Patients with Symptomatic Intracranial Stenosis. *Curr Neurovasc Res*. 2017;14:149-157. doi: 10.2174/1567202614666170413143203

44.	Leng X, Lan L, Ip HL, Abrigo J, Scalzo F, Liu H, Feng X, Chan KL, Fan FSY, Ma SH, et al. Hemodynamics and stroke risk in intracranial atherosclerotic disease. *Ann Neurol*. 2019;85:752-764. doi: 10.1002/ana.25456

45.	Fischer U, Hsieh-Meister K, Kellner-Weldon F, Galimanis A, Yan X, Kaesmacher J, El-Koussy M, Jung S, Arnold M, Michel P, et al. Symptomatic and asymptomatic intracranial atherosclerotic stenosis: 3 years' prospective study. *J Neurol*. 2020;267:1687-1698. doi: 10.1007/s00415-020-09750-2

46.	Feng X, Chan KL, Lan L, Abrigo J, Liu J, Fang H, Xu Y, Soo Y, Leng X, Leung TW. Stroke Mechanisms in Symptomatic Intracranial Atherosclerotic Disease: Classification and Clinical Implications. *Stroke*. 2019;50:2692-2699. doi: 10.1161/STROKEAHA.119.025732

47.	Kang DW, Kwon SU, Yoo SH, Kwon KY, Choi CG, Kim SJ, Koh JY, Kim JS. Early recurrent ischemic lesions on diffusion-weighted imaging in symptomatic intracranial atherosclerosis. *Arch Neurol*. 2007;64:50-54. doi: 10.1001/archneur.64.1.50

48.	Yaghi S, Mac Grory B, Prabhakaran S, Yeatts SD, Cutting S, Jayaraman M, Sacchetti D, Furie K, Zaidat OO, Liebeskind DS. Infarct pattern, perfusion mismatch thresholds, and recurrent cerebrovascular events in symptomatic intracranial stenosis. *Journal of Neuroimaging*. 2019;29:640-644.

49.	Kim JM, Jung KH, Sohn CH, Moon J, Shin JH, Park J, Lee SH, Han MH, Roh JK. Intracranial plaque enhancement from high resolution vessel wall magnetic resonance imaging predicts stroke recurrence. *Int J Stroke*. 2016;11:171-179. doi: 10.1177/1747493015609775

50.	Shi Z, Li J, Zhao M, Zhang X, Degnan AJ, Mossa-Basha M, Saloner D, Lu J, Liu Q, Zhu C. Progression of Plaque Burden of Intracranial Atherosclerotic Plaque Predicts Recurrent Stroke/Transient Ischemic Attack: A Pilot Follow-Up Study Using Higher-Resolution MRI. *J Magn Reson Imaging*. 2021;54:560-570. doi: 10.1002/jmri.27561

51.	Sangha RS, Naidech AM, Corado C, Ansari SA, Prabhakaran S. Challenges in the Medical Management of Symptomatic Intracranial Stenosis in an Urban Setting. *Stroke*. 2017;48:2158-2163. doi: 10.1161/STROKEAHA.116.016254

52.	Song X, Zhao X, Liebeskind DS, Wang L, Xu W, Xu Y, Hou D, Zheng Z, Wu J. Incremental value of plaque enhancement in predicting stroke recurrence in symptomatic intracranial atherosclerosis. *Neuroradiology*. 2020;62:1123-1131.

53.	Ssi-Yan-Kai G, Nasr N, Faury A, Catalaa I, Cognard C, Larrue V, Bonneville F. Intracranial artery stenosis or occlusion predicts ischemic recurrence after transient ischemic attack. *AJNR Am J Neuroradiol*. 2013;34:185-190. doi: 10.3174/ajnr.A3144

54.	Kate M, Sylaja PN, Kesavadas C, Thomas B. Imaging and clinical predictors of unfavorable outcome in medically treated symptomatic intracranial atherosclerotic disease. *J Stroke Cerebrovasc Dis*. 2014;23:973-978. doi: 10.1016/j.jstrokecerebrovasdis.2013.08.002

55.	Amin-Hanjani S, Pandey DK, Rose-Finnell L, Du X, Richardson D, Thulborn KR, Elkind MS, Zipfel GJ, Liebeskind DS, Silver FL. Effect of hemodynamics on stroke risk in symptomatic atherosclerotic vertebrobasilar occlusive disease. *JAMA neurology*. 2016;73:178-185.

56.	Chimowitz MI, Lynn MJ, Howlett-Smith H, Stern BJ, Hertzberg VS, Frankel MR, Levine SR, Chaturvedi S, Kasner SE, Benesch CG, et al. Comparison of warfarin and aspirin for symptomatic intracranial arterial stenosis. *N Engl J Med*. 2005;352:1305-1316. doi: 10.1056/NEJMoa043033

57.	Liu H, Pu Y, Wang Y, Zou X, Pan Y, Zhang C, Soo YO, Leung TW, Zhao X, Wong KSL. Intracranial atherosclerosis coexisting with white matter hyperintensities may predict unfavorable functional outcome in patients with acute cerebral ischemia. *Frontiers in Neurology*. 2020;11:609607.

58.	Shin DH, Lee PH, Bang OY. Mechanisms of recurrence in subtypes of ischemic stroke: a hospital-based follow-up study. *Arch Neurol*. 2005;62:1232-1237. doi: 10.1001/archneur.62.8.1232

59.	Thijs VN, Albers GW. Symptomatic intracranial atherosclerosis: outcome of patients who fail antithrombotic therapy. *Neurology*. 2000;55:490-497. doi: 10.1212/wnl.55.4.490

60.	Lange MC, Ribas G, Scavasine V, Ducci RD, Mendes DC, Zétola VHF, Cabral N, Rundek T. Stroke recurrence in the different subtypes of ischemic stroke. The importance of the intracranial disease. *Arq Neuropsiquiatr*. 2018;76:649-653. doi: 10.1590/0004-282X20180095

61.	Wong KS, Li H. Long-term mortality and recurrent stroke risk among Chinese stroke patients with predominant intracranial atherosclerosis. *Stroke*. 2003;34:2361-2366. doi: 10.1161/01.STR.0000089017.90037.7A

62.	Vaishnav AG, Vaishnav RA. Symptomatic Intracranial Atherosclerosis: Natural History of Medical Management Beyond the Hyperacute Stage of Ischemic Stroke in the Post-SAMMPRIS Era. *Journal of Stroke Medicine*. 2018;1:68-72.

63.	Tang CW, Chang FC, Chern CM, Lee YC, Hu HH, Lee IH. Stenting versus medical treatment for severe symptomatic intracranial stenosis. *AJNR Am J Neuroradiol*. 2011;32:911-916. doi: 10.3174/ajnr.A2409

64.	Ram R, Kaul S, Alladi S, Afshan JS, Prabha TS, Kohat A, Tandra S, Rani JY. Risk Factors, Vascular Lesion Distribution, Outcome and Recurrence of Strokes Due to Intracranial Atherosclerosis: One Year Data from Hyderabad Stroke Registry. *Ann Indian Acad Neurol*. 2017;20:387-392. doi: 10.4103/aian.AIAN_227_17

65.	Uehara T, Ohara T, Minematsu K, Nagatsuka K, Toyoda K. Predictors of Stroke Events in Patients with Transient Ischemic Attack Attributable to Intracranial Stenotic Lesions. *Intern Med*. 2018;57:295-300. doi: 10.2169/internalmedicine.9447-17

66.	Leung TW, Wang L, Soo YO, Ip VH, Chan AY, Au LW, Fan FS, Lau AY, Leung H, Abrigo J, et al. Evolution of intracranial atherosclerotic disease under modern medical therapy. *Ann Neurol*. 2015;77:478-486. doi: 10.1002/ana.24340

67.	Ilagan-Gacita SMQ, Sanchez-Tapia JAP, Navarro JC. Intracranial Arterial Stenosis Among Filipino Ischemic Stroke Patients: A Single Center Study. *Journal of Stroke Medicine*. 2020;3:131-137.

68.	Derdeyn CP, Chimowitz MI, Lynn MJ, Fiorella D, Turan TN, Janis LS, Montgomery J, Nizam A, Lane BF, Lutsep HL, et al. Aggressive medical treatment with or without stenting in high-risk patients with intracranial artery stenosis (SAMMPRIS): the final results of a randomised trial. *Lancet*. 2014;383:333-341. doi: 10.1016/S0140-6736(13)62038-3

69.	Zhou P, Lu Z, Gao P, Wang P, Cao Z, Zhang G, Wang S, Feng Y, Wang P. Efficacy and safety of intensive statin therapy in Chinese patients with atherosclerotic intracranial arterial stenosis: a single-center, randomized, single-blind, parallel-group study with one-year follow-up. *Clinical Neurology and Neurosurgery*. 2014;120:6-13.

70.	Arenillas JF, Álvarez-Sabín J, Molina CA, Chacón P, Montaner J, Rovira Á, Ibarra B, Quintana M. C-reactive protein predicts further ischemic events in first-ever transient ischemic attack or stroke patients with intracranial large-artery occlusive disease. *Stroke*. 2003;34:2463-2468.

71.	Suemoto CK, Grinberg LT, Leite REP, Ferretti-Rebustini REL, Jacob-Filho W, Yaffe K, Nitrini R, Pasqualucci CA. Morphometric measurements of extracranial and intracranial atherosclerotic disease: A population-based autopsy study. *Atherosclerosis*. 2018;270:218-223. doi: 10.1016/j.atherosclerosis.2017.12.015

72.	Qiao Y, Suri FK, Zhang Y, Liu L, Gottesman R, Alonso A, Guallar E, Wasserman BA. Racial Differences in Prevalence and Risk for Intracranial Atherosclerosis in a US Community-Based Population. *JAMA Cardiol*. 2017;2:1341-1348. doi: 10.1001/jamacardio.2017.4041

73.	Ahn JK, Hwang J, Hwang JH, Yoon WT, Chung PW, Ryu S. The association between serum uric acid and asymptomatic intracranial arterial stenosis in middle-aged Koreans. *Nutr Metab Cardiovasc Dis*. 2018;28:14-22. doi: 10.1016/j.numecd.2017.10.019

74.	Chen HX, Wang LJ, Yang Y, Yue FX, Chen LM, Xing YQ. The prevalence of intracranial stenosis in patients at low and moderate risk of stroke. *Ther Adv Neurol Disord*. 2019;12:1756286419869532. doi: 10.1177/1756286419869532

75.	Jin H, Peng Q, Nan D, Lv P, Liu R, Sun W, Teng Y, Liu Y, Fan C, Xing H, et al. Prevalence and risk factors of intracranial and extracranial artery stenosis in asymptomatic rural residents of 13 villages in China. *BMC Neurol*. 2017;17:136. doi: 10.1186/s12883-017-0924-0

76.	Kamal AK, Majeed F, Pasha O, Rehman H, Islam M, Azam I, Ilyas MS, Hussain M, Masood K, Ahmed B, et al. Clinical, lifestyle, socioeconomic determinants and rate of asymptomatic intracranial atherosclerosis in stroke free Pakistanis. *BMC Neurol*. 2014;14:155. doi: 10.1186/s12883-014-0155-6

77.	Kang K. Serum calcium and phosphate concentrations and intracranial atherosclerosis. *Atherosclerosis*. 2014;232:249-253. doi: 10.1016/j.atherosclerosis.2013.11.030

78.	Matsui R, Nakagawa T, Takayoshi H, Onoda K, Oguro H, Nagai A, Yamaguchi S. A Prospective Study of Asymptomatic Intracranial Atherosclerotic Stenosis in Neurologically Normal Volunteers in a Japanese Cohort. *Front Neurol*. 2016;7:39. doi: 10.3389/fneur.2016.00039

79.	Nam KW, Kwon HM, Jeong HY, Park JH, Kim SH, Jeong SM, Yoo TG, Kim S. Cerebral white matter hyperintensity is associated with intracranial atherosclerosis in a healthy population. *Atherosclerosis*. 2017;265:179-183. doi: 10.1016/j.atherosclerosis.2017.09.010

80.	Park KY, Chung CS, Lee KH, Kim GM, Kim YB, Oh K. Prevalence and risk factors of intracranial atherosclerosis in an asymptomatic korean population. *J Clin Neurol*. 2006;2:29-33. doi: 10.3988/jcn.2006.2.1.29

81.	Song X, Zhao Q, Hua Y, Wang C, Liu B, Guan S, Li J, Zhang Z, Fang X, Wu J. Association between blood pressure and intracranial artery stenosis in a Chinese population. *J Clin Hypertens (Greenwich)*. 2020;22:77-85. doi: 10.1111/jch.13770

82.	Leung SY, Ng TH, Yuen ST, Lauder IJ, Ho FC. Pattern of cerebral atherosclerosis in Hong Kong Chinese. Severity in intracranial and extracranial vessels. *Stroke*. 1993;24:779-786. doi: 10.1161/01.str.24.6.779

83.	Sun Q, Wang Q, Wang X, Ji X, Sang S, Shao S, Zhao Y, Xiang Y, Xue Y, Li J, et al. Prevalence and cardiovascular risk factors of asymptomatic intracranial arterial stenosis: the Kongcun Town Study in Shandong, China. *Eur J Neurol*. 2020;27:729-735. doi: 10.1111/ene.14144

84.	Wang A, Li Z, Luo Y, Liu X, Guo X, Wu S, Zhao X, Jonas JB. Asymptomatic intracranial arterial stenosis and metabolic syndrome: the APAC study. *PLoS One*. 2014;9:e113205.

85.	Wong KS, Huang YN, Yang HB, Gao S, Li H, Liu JY, Liu Y, Tang A. A door-to-door survey of intracranial atherosclerosis in Liangbei County, China. *Neurology*. 2007;68:2031-2034. doi: 10.1212/01.wnl.0000264426.63544.ee

86.	Zhai FF, Yan S, Li ML, Han F, Wang Q, Zhou LX, Ni J, Yao M, Zhang SY, Cui LY, et al. Intracranial Arterial Dolichoectasia and Stenosis: Risk Factors and Relation to Cerebral Small Vessel Disease. *Stroke*. 2018;49:1135-1140. doi: 10.1161/STROKEAHA.117.020130

87.	Shitara S, Fujiyoshi A, Hisamatsu T, Torii S, Suzuki S, Ito T, Arima H, Shiino A, Nozaki K, Miura K, et al. Intracranial Artery Stenosis and Its Association With Conventional Risk Factors in a General Population of Japanese Men. *Stroke*. 2019;50:2967-2969. doi: 10.1161/STROKEAHA.119.025964

88.	Gutierrez J, Elkind MS, Virmani R, Goldman J, Honig L, Morgello S, Marshall RS. A pathological perspective on the natural history of cerebral atherosclerosis. *Int J Stroke*. 2015;10:1074-1080. doi: 10.1111/ijs.12496

89.	Mazighi M, Labreuche J, Gongora-Rivera F, Duyckaerts C, Hauw JJ, Amarenco P. Autopsy prevalence of intracranial atherosclerosis in patients with fatal stroke. *Stroke*. 2008;39:1142-1147. doi: 10.1161/STROKEAHA.107.496513

90.	Zhou F, Tang J, Li P, Liao B, Qin C. Distribution of cerebral artery stenosis and risk factors in ethnic Zhuang and Han patients with ischemic stroke in Guangxi province. *Ann Palliat Med*. 2020;9:256-263. doi: 10.21037/apm.2020.02.32

91.	Ovesen C, Abild A, Christensen AF, Rosenbaum S, Hansen CK, Havsteen I, Nielsen JK, Christensen H. Prevalence and long-term clinical significance of intracranial atherosclerosis after ischaemic stroke or transient ischaemic attack: a cohort study. *BMJ Open*. 2013;3:e003724. doi: 10.1136/bmjopen-2013-003724

92.	Zhang S, Wang N, Chen L, Zhang P, Shen L, Huang S, Zhang C, Deng B. Serum Aldosterone Is Associated with Cerebral Artery Atherosclerosis and Calcification. *J Stroke Cerebrovasc Dis*. 2019;28:523-530. doi: 10.1016/j.jstrokecerebrovasdis.2018.09.053

93.	Ratanakorn D, Keandoungchun J, Tegeler CH. Coexistent extra- and intracranial stenosis, cervical atherosclerosis, and abnormal ankle brachial index in acute ischemic stroke. *J Stroke Cerebrovasc Dis*. 2012;21:782-789. doi: 10.1016/j.jstrokecerebrovasdis.2011.04.006

94.	Barreto-Neto N, Barros AD, Jesus PA, Reis CC, Jesus ML, Ferreira IL, Fernandes RD, Resende LL, Andrade AL, Gonçalves BM, et al. Low Ankle-Brachial Index is a Simple Physical Exam Sign Predicting Intracranial Atherosclerotic Stenosis in Ischemic Stroke Patients. *J Stroke Cerebrovasc Dis*. 2016;25:1417-1420. doi: 10.1016/j.jstrokecerebrovasdis.2016.01.049

95.	Lee SJ, Lee DG, Lim DS, Hong S. Impact of Intracranial Atherosclerotic Stenosis on the Prognosis in Acute Ischemic Stroke Patients with Cardioembolic Source. *Eur Neurol*. 2015;73:271-277. doi: 10.1159/000381336

96.	Palacio S, McClure LA, Benavente OR, Bazan C, Pergola P, Hart RG. Lacunar strokes in patients with diabetes mellitus: risk factors, infarct location, and prognosis: the secondary prevention of small subcortical strokes study. *Stroke*. 2014;45:2689-2694. doi: 10.1161/STROKEAHA.114.005018

97.	Leng X, Hurford R, Feng X, Chan KL, Wolters FJ, Li L, Soo YO, Wong KSL, Mok VC, Leung TW, et al. Intracranial arterial stenosis in Caucasian versus Chinese patients with TIA and minor stroke: two contemporaneous cohorts and a systematic review. *J Neurol Neurosurg Psychiatry*. 2021. doi: 10.1136/jnnp-2020-325630

98.	Hua Y, Jia L, Xing Y, Hui P, Meng X, Yu D, Pan X, Fang Y, Song B, Wu C, et al. Distribution Pattern of Atherosclerotic Stenosis in Chinese Patients with Stroke: A Multicenter Registry Study. *Aging Dis*. 2019;10:62-70. doi: 10.14336/AD.2018.0602

99.	Kim J, Heo JH. Response to letter regarding article, "Serum alkaline phosphatase and phosphate in cerebral atherosclerosis and functional outcomes after cerebral infarction". *Stroke*. 2014;45:e47. doi: 10.1161/STROKEAHA.113.004281

100.	Guo J, Su W, Fang J, Chen N, Zhou M, Zhang Y, He L. Elevated CRP at admission predicts post-stroke cognitive impairment in Han Chinese patients with intracranial arterial stenosis. *Neurol Res*. 2018;40:292-296. doi: 10.1080/01616412.2018.1438224

101.	Park HK, Kim BJ, Han MK, Park JM, Kang K, Lee SJ, Kim JG, Cha JK, Kim DH, Nah HW, et al. One-Year Outcomes After Minor Stroke or High-Risk Transient Ischemic Attack: Korean Multicenter Stroke Registry Analysis. *Stroke*. 2017;48:2991-2998. doi: 10.1161/STROKEAHA.117.018045

102.	Park JH, Chung JW, Bang OY, Kim GM, Choi KH, Park MS, Kim JT, Hwang YH, Song TJ, Kim YJ, et al. Atherosclerotic Burden and Vascular Risk in Stroke Patients With Atrial Fibrillation. *Stroke*. 2021;52:1662-1672. doi: 10.1161/STROKEAHA.120.032232

103.	Park JH, Kwon HM, Lee J, Kim DS, Ovbiagele B. Association of intracranial atherosclerotic stenosis with severity of white matter hyperintensities. *Eur J Neurol*. 2015;22:44-52, e42-43. doi: 10.1111/ene.12431

104.	Park JH, Kwon HM, Roh JK. Metabolic syndrome is more associated with intracranial atherosclerosis than extracranial atherosclerosis. *Eur J Neurol*. 2007;14:379-386. doi: 10.1111/j.1468-1331.2007.01682.x

105.	Li H, Wong KS, Kay R. Relationship between the Oxfordshire Community Stroke Project classification and vascular abnormalities in patients with predominantly intracranial atherosclerosis. *J Neurol Sci*. 2003;207:65-69. doi: 10.1016/s0022-510x(02)00397-0

106.	Uchiyama S, Hoshino T, Sissani L, Linsay MT, Kamiyama K, Nakase T, Kitagawa K, Minematsu K, Todo K, Okada Y, et al. Japanese Versus Non-Japanese Patients with Transient Ischemic Attack or Minor Stroke: Subanalysis of TIA registry.org. *J Stroke Cerebrovasc Dis*. 2019;28:2232-2241. doi: 10.1016/j.jstrokecerebrovasdis.2019.05.005

107.	Zhang CY, Huang SR, Wang SY, Shen L, Deng BQ. Clinical Study of Intracranial and Extracranial Atherosclerotic Stenosis in Spontaneous Intracerebral Hemorrhage Patients. *J Stroke Cerebrovasc Dis*. 2018;27:286-290. doi: 10.1016/j.jstrokecerebrovasdis.2017.08.011

108.	Kamtchum-Tatuene J, Mwandumba HC, Mwangalika Kachingwe G, Bonnett LJ, Kayange N, Solomon T, Benjamin LA. A cross-sectional feasibility study of neurovascular ultrasound in Malawian adults with acute stroke-like syndrome. *PLoS One*. 2020;15:e0229033. doi: 10.1371/journal.pone.0229033

109.	De Silva DA, Woon FP, Lee MP, Chen CL, Chang HM, Wong MC. Metabolic syndrome is associated with intracranial large artery disease among ethnic Chinese patients with stroke. *J Stroke Cerebrovasc Dis*. 2009;18:424-427. doi: 10.1016/j.jstrokecerebrovasdis.2009.01.010

110.	Viaro F, Farina FM, Onofri A, Meneghetti G, Baracchini C. Symptomatic intracranial stenosis: a university hospital-based ultrasound study. *Perspectives in Medicine*. 2012;1:211-213.

111.	Von Sarnowski B, Schminke U, Tatlisumak T, Putaala J, Grittner U, Kaps M, Tobin WO, Kinsella JA, McCabe DJ, Hennerici MG. Prevalence of stenoses and occlusions of brain-supplying arteries in young stroke patients. *Neurology*. 2013;80:1287-1294.

112.	Li MM, Lin YY, Huang YH, Zhuo ST, Yang ML, Lin HS, Cai RW. Association of Apolipoprotein A1, B with Stenosis of Intracranial and Extracranial Arteries in Patients with Cerebral Infarction. *Clin Lab*. 2015;61:1727-1735. doi: 10.7754/clin.lab.2015.150419

113.	Tawfik TZ, Zaki MA, Abd Allah FA, Farrag MA, Auis EB, Hegazy MM. The distribution of atherosclerosis in Egyptian patients with ischemic stroke: extracranial versus intracranial atherosclerotic carotid artery disease; ultrasonographic assessment. *Egypt J Neurol Psychiat Neurosurg*. 2009;46:279-285.

114.	Wang YL, Leng XY, Dong Y, Hou XH, Tong L, Ma YH, Xu W, Cui M, Dong Q, Tan L, et al. Fasting glucose and HbA. *Ann Transl Med*. 2019;7:804. doi: 10.21037/atm.2019.12.56

115.	Markus HS, Khan U, Birns J, Evans A, Kalra L, Rudd AG, Wolfe CD, Jerrard-Dunne P. Differences in stroke subtypes between black and white patients with stroke: the South London Ethnicity and Stroke Study. *Circulation*. 2007;116:2157-2164. doi: 10.1161/CIRCULATIONAHA.107.699785

116.	Moussouttas M, Aguilar L, Fuentes K, Anyanwu B, Manassarians H, Papamitsakis N, Shi Q, Visintainer P. Cerebrovascular disease among patients from the Indian subcontinent. *Neurology*. 2006;67:894-896. doi: 10.1212/01.wnl.0000233923.63869.8c

117.	Meseguer E, Lavallée PC, Mazighi M, Labreuche J, Cabrejo L, Olivot JM, Abboud H, Slaoui T, Lapergue B, Guidoux C, et al. Yield of systematic transcranial Doppler in patients with transient ischemic attack. *Ann Neurol*. 2010;68:9-17. doi: 10.1002/ana.21921

118.	Shi Z, Zhang X, Chen Z, Liebeskind DS, Lou M. Elevated thyroid autoantibodies and intracranial stenosis in stroke at an early age. *International Journal of Stroke*. 2014;9:735-740.

119.	Kim YD, Choi HY, Cho HJ, Cha MJ, Nam CM, Han SW, Nam HS, Heo JH. Increasing frequency and burden of cerebral artery atherosclerosis in Korean stroke patients. *Yonsei medical journal*. 2010;51:318-325.

120.	Hoshi T, Kitagawa K, Yamagami H, Furukado S, Hougaku H, Hori M. Relation between interleukin-6 level and subclinical intracranial large-artery atherosclerosis. *Atherosclerosis*. 2008;197:326-332. doi: 10.1016/j.atherosclerosis.2007.05.013

121.	Suri MF, Qiao Y, Ma X, Guallar E, Zhou J, Zhang Y, Liu L, Chu H, Qureshi AI, Alonso A, et al. Prevalence of Intracranial Atherosclerotic Stenosis Using High-Resolution Magnetic Resonance Angiography in the General Population: The Atherosclerosis Risk in Communities Study. *Stroke*. 2016;47:1187-1193. doi: 10.1161/STROKEAHA.115.011292

122.	Bae HJ, Lee J, Park JM, Kwon O, Koo JS, Kim BK, Pandey DK. Risk factors of intracranial cerebral atherosclerosis among asymptomatics. *Cerebrovasc Dis*. 2007;24:355-360. doi: 10.1159/000106982

123.	Park SY, An SA, Lee HB, Kim Y, Kim NK, Kim SH, Kim OJ, Oh SH. Different impact of hyperhomocysteinemia on cerebral small vessel ischemia and cervico-cerebral atherosclerosis in non-stroke individuals. *Thromb Res*. 2013;131:e12-16. doi: 10.1016/j.thromres.2012.11.011

124.	Liu B, Zhou F, Hua Y, Liu Y, Ji X. Evaluation of intracranial and extracranial atherosclerotic lesions in patients with symptomatic coronary artery disease. *Neurol Res*. 2020;42:547-553. doi: 10.1080/01616412.2020.1754653

125.	Jellinger KA, Attems J. Neuropathology and general autopsy findings in nondemented aged subjects. *Clin Neuropathol*. 2012;31:87-98. doi: 10.5414/np300418

126.	Alkan O, Kizilkilic O, Yildirim T, Atalay H. Intracranial cerebral artery stenosis with associated coronary artery and extracranial carotid artery stenosis in Turkish patients. *Eur J Radiol*. 2009;71:450-455. doi: 10.1016/j.ejrad.2008.05.006

127.	Li Z, Li J, Wang A, Pan H, Wu S, Zhao X. Decreased Estimated Glomerular Filtration Rate (eGFR), Not Proteinuria, Is Associated with Asymptomatic Intracranial Arterial Stenosis in Chinese General Population. *Sci Rep*. 2017;7:4619. doi: 10.1038/s41598-017-04549-0

128.	Zwartbol MHT, Geerlings MI, Ghaznawi R, Hendrikse J, van der Kolk AG, Group U-SS. Intracranial Atherosclerotic Burden on 7T MRI Is Associated with Markers of Extracranial Atherosclerosis: The SMART-MR Study. *AJNR Am J Neuroradiol*. 2019;40:2016-2022. doi: 10.3174/ajnr.A6308

129.	López-Cancio E, Dorado L, Millán M, Reverté S, Suñol A, Massuet A, Galán A, Alzamora MT, Pera G, Torán P, et al. The Barcelona-Asymptomatic Intracranial Atherosclerosis (AsIA) study: prevalence and risk factors. *Atherosclerosis*. 2012;221:221-225. doi: 10.1016/j.atherosclerosis.2011.12.020

130.	Kim YS, Hong JW, Jung WS, Park SU, Park JM, Cho SI, Bu YM, Moon SK. Gender differences in risk factors for intracranial cerebral atherosclerosis among asymptomatic subjects. *Gend Med*. 2011;8:14-22. doi: 10.1016/j.genm.2011.01.001

131.	Modesti PA, Castellani S, Calabrese M, Malandrino D, Zhao D. Comparison of type 2 diabetes prevalence in Chinese migrants vs Caucasians and new perspectives for screening of cerebrovascular disease in Chinese: A proof of concept study. *Diabetes Res Clin Pract*. 2017;130:196-203. doi: 10.1016/j.diabres.2017.05.023

132.	Abd-Allah F, Ibrahim EM, Zidan O, Mohamed MA, Mohamdy AA, Farrag MA, Aboulfotooh AM, Gomaa HA. Screening of Asymptomatic Intracranial Arterial Stenosis among High Risk Subjects: A Pilot Study from Egypt. *J Vasc Interv Neurol*. 2018;10:68-72.

133.	Abd-Allah F, Kassem HH, Hashad A, Shamloul RM, Zaki A. Prevalence of intracranial atherosclerosis among patients with coronary artery disease: a 1-year hospital-based study. *Eur Neurol*. 2014;71:326-330. doi: 10.1159/000358055

134.	Lee SJ, Cho SJ, Moon HS, Shon YM, Lee KH, Kim DI, Lee BB, Byun HS, Han SH, Chung CS. Combined extracranial and intracranial atherosclerosis in Korean patients. *Arch Neurol*. 2003;60:1561-1564. doi: 10.1001/archneur.60.11.1561

135.	Oh HG, Chung PW, Rhee EJ. Increased risk for intracranial arterial stenosis in subjects with coronary artery calcification. *Stroke*. 2015;46:151-156. doi: 10.1161/STROKEAHA.114.006996

136.	Valaikiene J, Ryliskyte L, Valaika A, Puronaite R, Dementaviciene J, Vaitkevicius A, Badariene J, Butkuviene I, Kalinauskas G, Laucevicius A. A High Prevalence of Intracranial Stenosis in Patients with Coronary Artery Disease and the Diagnostic Value of Transcranial Duplex Sonography. *J Stroke Cerebrovasc Dis*. 2019;28:1015-1021. doi: 10.1016/j.jstrokecerebrovasdis.2018.12.023

137.	Elmore EM, Mosquera A, Weinberger J. The prevalence of asymptomatic intracranial large-vessel occlusive disease: the role of diabetes. *J Neuroimaging*. 2003;13:224-227.

138.	Salem HH, El-Deen H, Al-Azzouny A, Saleh A, Abdulghani M, Etribi M. Cerebral atherosclerosis in ischemic heart disease. *Egypt J Neurol Psychiatry Neurosurg*. 2010;47:647-654.

139.	Elwan ME, El Sheikh WM, El Kabany RA, Nirmeen K, Soliman HS. Intracranial Atherosclerosis in Type II Diabetics and Correlation with Cognitive Impairment. *Egyptian Journal of Neurology, Psychiatry & Neurosurgery*. 2014;51.
